# HDAC1 maintains metabolic homeostasis in nucleus pulposus cells via post-translational modification interactions with YBX1

**DOI:** 10.1038/s12276-026-01843-8

**Published:** 2026-09-09

**Authors:** Bohao Zhou, Jiechao Xia, Zhaowei Zhang, Bingbing Xu, Zixin Ruan, Tianyou Gao, Chen’an You, Fengjie Ma, Zhenlei Zhang, Teng Yao, Jun Gao, Jianle Wang, Zheyuan Zhang, Junxin Chen, Shiyuan Shi, Yongfeng Cui, Panyang Shen, Shuying Shen, Jianjun Ma

**Affiliations:** 1 Department of Orthopaedic Surgery, Sir Run Run Shaw Hospital, Zhejiang University School of Medicine, Hangzhou, China; 2 School of Medicine, Shaoxing University, Shaoxing, Zhejiang China; 3 School of Basic Medical Sciences and Forensic Medicine, Hangzhou Medical College, Hangzhou, China; 4 Department of Orthopaedic Surgery, Hangzhou Ninth People’s Hospital, Hangzhou, China

**Keywords:** Cartilage, Acetylation

## Abstract

Intervertebral disc degeneration is characterized by the metabolic dysregulation of nucleus pulposus cells, which is regulated by complex post-translational modifications, manifesting as extracellular matrix remodeling and inflammatory microenvironment formation. We demonstrated the progressive downregulation of *HDAC1* during disc degeneration. HDAC1 mediates the deacetylation and ubiquitination-dependent degradation of YBX1. The downregulation of *HDAC1* results in YBX1 accumulation, compromising extracellular matrix synthesis in nucleus pulposus cells while enhancing the degradation processes. Furthermore, the accumulated YBX1 stimulates chemokine expression, facilitating the development of an inflammatory microenvironment. Targeting this critical axis, we developed SU056@GelMA to deliver the YBX1 inhibitor SU056, mitigating the metabolic disruption induced by YBX1 accumulation.

## HDAC1 downregulation drives inflammation in disc degeneration

Intervertebral disc degeneration (IVDD) is a leading cause of disability, with nucleus pulposus cells (NPCs) undergoing metabolic changes that lead to extracellular matrix (ECM) degradation and inflammation. This study explores the role of histone deacetylase 1 (HDAC1) in IVDD, revealing its downregulation in degenerative discs and its critical role in maintaining NPC metabolic homeostasis. Researchers used RNA sequencing and protein analysis to demonstrate that HDAC1 regulates ECM synthesis and degradation by deacetylating Y-box-binding protein 1 (YBX1), promoting its ubiquitination and degradation. HDAC1 deficiency led to YBX1 accumulation, promoting ECM degradation and inflammation. The study introduced SU056@GelMA, a hydrogel delivering the YBX1 inhibitor SU056, which improved ECM synthesis and reduced inflammation in rat and rabbit IVDD models. These findings highlight the potential of HDAC1 as a therapeutic target, suggesting future research on post-translational modifications in IVDD treatment. "This summary was initially drafted using artificial intelligence, then revised and fact-checked by the author. "

## Introduction

Low back pain is the primary cause of disability worldwide^1^, and intervertebral disc degeneration (IVDD) is emerging as its predominant etiological factor^2^. During IVDD progression, nucleus pulposus cells (NPCs) undergo metabolic perturbations characterized by extensive extracellular matrix (ECM) remodeling and inflammatory microenvironment formation^3,4^. Post-translational modifications (PTMs) serve as critical regulators of IVDD pathogenesis^5^. For example, once phosphorylated, 5′ adenosine monophosphate-activated protein kinase inhibits mammalian target of rapamycin phosphorylation and induces Unc-51 like autophagy activating kinase 1 phosphorylation, thereby enhancing autophagic activity in NPCs^6,7^. Furthermore, ataxia telangiectasia and Rad3-related protein deficiency reduces tripartite motif-containing 56 protein binding shifting its ubiquitination from K63 to K48, promotes ataxia telangiectasia and Rad3-related protein degradation, and compromises genomic stability^8^.

Acetylation, a critical PTM, is regulated by histone acetyltransferases and histone deacetylases (HDACs)^9^. Traditionally, HDACs regulate transcription by deacetylating histones, enhancing DNA–histone interactions, and preventing transcription factor binding^10,11^. Recent studies have highlighted the importance of non-histone acetylation in degenerative diseases. In chondrocytes, a stiff ECM downregulates *HDAC3*, causing Parkin acetylation and excessive mitophagy, thereby accelerating senescence and osteoarthritis^12^. Conversely, sirtuin 6 attenuates chondrocyte senescence by deacetylating signal transducer and activator of transcription 5 and inhibiting its nuclear translocation^13^; however, our understanding of non-histone acetylation in IVDD remains limited.

PTM enzymes can modify each other, whereas substrates may contain multiple modification sites, creating a crosstalk between different modifications^14^. Acetylation acts as a switch to regulate other protein modifications through dynamic acetylation/deacetylation, thereby controlling protein activity and stability^15,16^. For example, in the regulation of myeloid cell leukemia 1, acetylation decreases its phosphorylation and ubiquitination and increases stability, whereas deacetylation produces opposite effects^17^. In chondrocytes, HDAC3 deacetylates and subsequently deubiquitinates fatty acid synthase, enhancing its stability^18^. Single-cell sequencing identifies chondrocyte-like NPCs as the predominant subpopulation within NPCs, suggesting that NPCs share metabolic characteristics with chondrocytes^19,20^. We hypothesized that the interactions between PTMs constitute critical components of this dynamic process during NPC degeneration and metabolic network remodeling.

In this study, we combined publicly available RNA sequencing (RNA-seq) data with *HDAC1* expression analysis in human nucleus pulposus (NP) tissue samples and found that *HDAC1* was downregulated during IVDD progression. As HDAC1 functions as a deacetylase, we hypothesized that it might influence the function of NPCs by targeting the PTM networks of specific substrate proteins. Therefore, we investigated the effect of HDAC1 on NPC metabolism, focusing on its role as a deacetylase.

## Materials and methods

### Human NP tissue samples

Human NP tissue samples were obtained from patients who underwent vertebral fusion surgery for lumbar disc herniation, spinal stenosis, or vertebral fractures. The study protocol was approved by the Ethics Committee of Sir Run Run Shaw Hospital, Zhejiang University School of Medicine, and comprehensive informed consent was obtained from all participants. Using magnetic resonance imaging (MRI) and the Pfirrmann grading system^21^, patients were stratified into four distinct groups: grade II (*n *= 7, mean age 51 years), grade III (*n *= 10, mean age 49 years), grade IV (*n *= 13, mean age 58 years), and grade V (*n *= 5, mean age 57 years). The NP samples were washed with sterile phosphate-buffered saline (PBS) until the supernatant was clear. Subsequent processing involved embedding, sectioning, and application of multiple histological staining techniques, including hematoxylin and eosin (HE), safranin O and fast green (SO & FG), and Alcian Blue staining.

### Cell culture

Surgically excised NP samples were washed with PBS to ensure optimal transparency. Employing ophthalmic precision instruments, the tissue was separated into approximately 1 mm^3^ specimens. The fragmented NP tissue was transferred to T75 culture flasks, immersed in type II collagenase solution, and subjected to overnight incubation at 37 °C with 5% CO_2_ atmospheric conditions. The subsequent digestion process involved filtering the liquid through a 100-μm cell strainer (BS-100-XBS), centrifuging at 1000 rpm for 5 min, and resusupending the cell pellet in Dulbecco’s modified Eagle’s medium (DMEM) supplemented with 10% fetal bovine serum (Cellmax, SA301.02). Next, 9 ml of 10% DMEM containing the cell suspension was distributed into a new T75 flask, thoroughly mixed, and incubated. Consistent maintenance protocols were implemented, involving washing with PBS and replacing the medium until optimal cell confluence was achieved.

Using CO_2_ euthanasia, 4-week-old male Sprague–Dawley rats were obtained from the Zhejiang University Animal Experimental Center (approved by the Institutional Ethics Committee). The rat tail vertebrae were carefully extracted, and the surrounding muscle and connective tissues were removed to expose the intervertebral disc. Following disc excision, the gelatinous NP tissue was isolated using ophthalmic scissors and forceps and subjected to PBS washing via triple centrifugation cycles.

The NP tissue was then transferred to T75 culture flasks for type II collagenase digestion for 4 h at 37 °C with 5% CO_2_. After digestion, filtration, centrifugation, and cell pellet resuspension in DMEM medium supplemented with 10% fetal bovine serum were performed according to standard protocols. Consistent with human NPC culture, maintenance involved bidaily PBS washing and medium replacement until complete cell confluence^22^.

### Gene knockout and overexpression

Upon reaching confluence, NPCs were passaged at a ratio of 1:3 into six-well plates. Once 60–70% confluent, NPCs were transfected with siRNA (0.08 OD per well) using Lipofectamine RNAiMAX Transfection Reagent (Invitrogen, 13778150). A standardized protocol was employed: medium replacement after 24 h, followed by RNA and protein collection at 48 h, or cell digestion, enumeration, and 24-well plate fixation.

Using the Hieff Trans Liposomal Transfection Reagent (Yeasen, 40802ES03), HEK293T cells were transfected with HA–HDAC1/HA–VECTOR/FLAG–YBX1/FLAG–VECTOR alongside pMD2.G and psPAX2. After medium replacement and centrifugation of the viral supernatant, the viral supernatant was mixed with polybrene at a virus-to-polybrene ratio of 1000:1 (v/v) and subsequently added to NPCs. Exogenous protein expression was achieved through 24-h puromycin (2 μg/ml) selection^23^.

### Western blot

NPCs were subjected to comprehensive lysis using PKILL solution supplemented with phenylmethylsulfonyl fluoride. The protein solution was collected, combined with loading buffer (Fdbio, FD002), and thermally denatured at 100 °C for 10 min. Proteins were separated by sodium dodecyl sulfate polyacrylamide gel electrophoresis (SDS–PAGE; initial 60 V for 30 min, and then 130 V for 80 min), followed by transfer to a polyvinylidene fluoride membrane (BIO-RAD, 1620177) at 200 mA for 120 min. Following 5% skimmed milk blocking, the membrane was incubated overnight at 4 °C with primary antibodies encompassing β-actin (1:20000, Proteintech, 66009-1-Ig), MMP3 (1:1000, Abcam, ab52915), ADAMTS5 (1:1000, Abcam, ab41037), SOX9 (1:1000, Abcam, ab185230), COL2A1 (1:1000, Abcam, ab34712), HDAC1 (1:1000, Abcam, ab280198), YBX1 (1:1000, Abcam, ab255606), UB (1:1000, Proteintech, 10201-2-AP), or ACE-LYS (1:1000, Cell Signaling, #9441). Secondary antibody incubation with diluted mouse/rabbit horseradish peroxidase-conjugated variants preceded protein band visualization using enhanced chemiluminescence exposure solution and chemiluminescence imaging (VILBER, FUSION-FX6. EDGE V0.070). Quantitative analysis was performed using the ImageJ software.

### RNA extraction, RT-qPCR, and RNA-seq

NPCs were lysed using TRIzol reagent (AG, AG21102) and subsequently subjected to RNA extraction using a SteadyPure RNA Extraction Kit (AG, AG21024). The RNA concentration was determined spectrophotometrically, followed by reverse transcription using dedicated reagents (AG, AG11706). Quantitative polymerase chain reaction (qPCR) was performed using SYBR Green (Yeasen, 11201ES03) on a real-time fluorescence qPCR system (Thermo, A28140) using the primer sequences detailed in Supplementary Table 2. Subsequent data analysis was conducted using Microsoft Excel and GraphPad Prism^24^.

*YBX1* was knocked down in human NPCs using si-*YBX1* (comprising control and knockdown groups, each incorporating three biological replicates). Cellular lysis was performed 48 h after transfection using TRIzol, with samples subsequently sent to Majorbio Bio-Pharm Technology (Shanghai) for comprehensive RNA-seq analysis. Sophisticated bioinformatics analysis and visualization were executed using Majorbio Cloud Platform (www.MajorBio.com), Bioinformatics (https://www.bioinformatics.com.cn)^25^, and OmicStudio tools (https://www.omicstudio.cn/tool)^26^.

### IF and IHC

Paraffin sections were subjected to systematic deparaffinization and antigen retrieval, followed by endogenous enzyme quenching using 3% hydrogen peroxide. Cell slides or paraffin sections were blocked uniformly with 5% bovine serum albumin for a standardized 1-h duration. Primary antibodies were diluted to 1:200 concentrations, including MMP3 (Proteintech, 17873-1-AP), ADAMTS5 (Abcam, ab41037), SOX9 (Proteintech, 67439-1-Ig), COL2A1 (Proteintech, 28459-1-AP), HDAC1 (Proteintech, 10197-1-AP), YBX1 (Proteintech, 20339-1-AP), CD68 (Proteintech, 25747-1-AP), CD80/B7-1 (Proteintech, 66406-1-Ig), CD206/MRC1 (Proteintech, 18704-1-AP), MPO (Proteintech, 66177-1-Ig), and ELA2 (Proteintech, 27642-1-AP), and incubated overnight at 4°C. Cell slides were incubated with appropriately diluted mouse/rabbit fluorescent secondary antibodies (Fdbio, FD0156, FD0143, FD0150, and FD0161). DAPI solution (1:1000 dilution, Solarbio, C0060) was subsequently applied to visualize nuclei, and the slides were mounted using an antifade mounting medium (Beyotime, P0126) and imaged via fluorescence microscopy. Immunohistochemistry (IHC) slides were incubated with polymer horseradish peroxidase-labeled rabbit/mouse IgG, developed with 3,3′-diaminobenzidine chromogenic solution, mounted with neutral resin, and imaged through bright-field microscopy.

### Protein MS and co-IP

Rat NPCs were transfected with FLAG–Hdac1 via lentiviral transduction, and subsequent cellular lysis was performed using a weak radioimmunoprecipitation assay (RIPA) buffer. Crosslinked anti-FLAG antibody or IgG magnetic beads were incubated with the lysate. After magnetic separation and rigorous washing, the beads were sent to Shanghai Shice Biotechnology Co., Ltd.for comprehensive protein mass spectrometry (MS) analysis.

HEK293T cells were transfected with HA–HDAC1/HA–VECTOR/FLAG–YBX1/FLAG–VECTOR plasmids to facilitate exogenous protein expression. Cellular lysis was performed using a weak RIPA solution (FD011; Fdbio). FLAG (Proteintech, 66008-4-Ig) and HA (Proteintech, 66006-2-Ig) antibodies were systematically crosslinked to A/G magnetic beads (Yeasen, 36417ES03). The lysate was then mixed with crosslinked antibody-conjugated beads and incubated overnight at 4 °C. Protein denaturation and elution were accomplished using a loading buffer, followed by western blotting analysis.

### Animal experiments

*Hdac1* knockout (KO) mice were procured from Cyagen (Supplementary Fig. 4a). The mice were subjected to systematic breeding and genotyping. Subsequently, ten male *Hdac1* wildtype (WT) and ten male *Hdac1* KO mice were stratified into four experimental groups: WT SHAM, WT IVDD, KO SHAM, and KO IVDD. The WT SHAM and KO SHAM groups received anesthesia using a combination of Zoletil 50 (40 mg/kg) and xylazine (5 mg/kg) without additional intervention. For the WT and KO IVDD groups, following anesthesia, a precision 29 G needle was carefully inserted into the mouse tail intervertebral space. At 4 weeks postprocedural intervention, the mice were euthanized, specimens were fixed using paraformaldehyde, X-ray imaging was performed, and the samples were processed through decalcification, embedding, and sectioning. Histological characterization was performed using HE, SO & FG, and Alcian Blue staining.

Six-week-old male rats were anesthetized using a combination of Zoletil 50 (15 mg/kg) and xylazine (5 mg/kg) with a 26 G needle precisely inserted into the rat tail intervertebral space, rotated circumferentially once, and withdrawn after a standardized 1-min interval. Employing a microinjection syringe, 20 μl of PBS solution, gelatin methacryloyl (GelMA), or SU056@GelMA was injected into the intervertebral disc, establishing distinct PBS injection, GelMA, and SU056@GelMA experimental groups. Following a 4-week observation period, the rats were euthanized, specimens were collected and fixed with paraformaldehyde, X-ray images were captured, and the samples were processed through decalcification, embedding, and sectioning. Histological characterization was conducted using HE, SO & FG, and Alcian Blue staining.

Six-month-old New Zealand White (NZW) rabbits were randomly assigned to SHAM, Puncture + PBS, GelMA, or SU056@GelMA groups (*n *= 5 per group). Animals were anesthetized intramuscularly with Zoletil 50 (20 mg/kg) and xylazine (5 mg/kg). The target lumbar intervertebral disc was exposed through a posterolateral approach with blunt dissection along the psoas–erector spinae interval. In the SHAM group, the disc was exposed without puncture or intradiscal injection. In the remaining groups, IVDD was induced by inserting a 29 G needle into the target disc, rotating the needle circumferentially once, and retaining it for 1 min^27^. Subsequently, 20 μl of PBS, GelMA, or SU056@GelMA was injected intradiscally using a microsyringe according to group allocation; the GelMA and SU056@GelMA groups received immediate 405 nm light irradiation for 1 min to induce photocrosslinking. The wounds were closed in layers, and the same intradiscal injection and photocrosslinking procedure was repeated at week 2.

At week 6, rabbits were euthanized intravenously with sodium pentobarbital (100 mg/kg), and the operated lumbar segments were harvested for X-ray imaging, fixation, decalcification, paraffin embedding, and transverse sectioning. Histological evaluation was performed using HE, SO & FG, and Alcian Blue staining^28^. IF/IHC analyses were conducted for matrix metabolism and inflammatory cell infiltration markers, including COL2A1, MMP3, CXCL8, CD68/CD80, and MPO/ELA2, according to the protocols described above.

All animal experiments were approved by the Zhejiang University Laboratory Animal Welfare and Ethics Review Committee (approval Nos. ZJU20250408 for mouse/rat experiments and ZJU20260485 for NZW rabbit experiments) and were conducted in accordance with relevant guidelines and regulations, including the National Research Council’s Guide for the Care and Use of Laboratory Animals. Specific pathogen-free male animals were used as follows: C57-background mice (8 weeks old, approximately 40 g; total *n* = 20; Hdac1 KO mice from Cyagen as described above), Sprague–Dawley rats (6 weeks old, approximately 120 g; total *n* = 35; Zhejiang University Laboratory Animal Center), and NZW rabbits (6 months old, approximately 2.5 kg; total *n* = 20; Hangzhou Yuhang Kelian Rabbit Industry Professional Cooperative, SCXK (Zhe) 2022-0008)^29^.

### Synthesis and characterization of SU056@GelMA

GelMA and lithium phenyl-2,4,6-trimethylbenzoylphosphinate (LAP) were acquired from EFL. The initial preparation involved the generation of 5% and 10% GelMA solutions. The requisite GelMA solid mass was combined with 0.25% LAP initiator, heated at 70 °C in a light-protected environment for 25 min to ensure complete dissolution, and centrifuged at 3000 rpm for 2 min to eliminate potential bubbles. The solution was immediately sterilized through a 0.22 μm filter and crosslinked under 405 nm UV light for 1 min. Individual samples were stored at −80 °C and subjected to overnight freeze-drying. The samples were mounted on a tape, gold-sputtered for 1 min, and comprehensively examined and photographed using a scanning electron microscope (SU-8010)^30^.

### GelMA hydrogel swelling rate, degradation rate, and mechanical properties

The swelling rate was assessed using the gravimetric methodology. Cylindrical GelMA specimens (10 mm diameter, 5 mm height) crosslinked under 405 nm UV light were freeze-dried, immersed in PBS solution, and incubated at 37 °C until predetermined time points. The surface liquid was removed, and the swelling rate was calculated using the formula Wt / W0 × 100%, where W0 represents the initial sample mass and Wt represents the mass at a specific time point.

Hydrogel stability was systematically evaluated using a weight-based approach. Cylindrical GelMA specimens (10 mm diameter, 5 mm height) were soaked in sterile PBS at 37 °C for multiple weeks. The surface liquid was removed and the samples were weighed at predetermined time points. The remaining mass percentage was calculated using the formula Wt / W0 × 100%, where W0 represents the initial sample mass and Wt represents the mass at a specific time point.

The mechanical properties were comprehensively assessed using tensile tests. Cylindrical GelMA specimens (10 mm length, 10 mm diameter) crosslinked under 405 nm UV light were secured at one end and subjected to controlled pulling at the opposite end at a speed of 1 mm/min using a universal testing machine^31^.

### SU056@GelMA release performance analysis

To quantitatively characterize SU056 metabolism in vivo, CY3–COOH dye was incorporated into SU056 (Xi’an Qiyue Biotechnology). The synthesized CY3-labeled SU056 exhibited fluorescence emission at 570 nm upon excitation at 550 nm.

PBS (2 ml) at pH 7.4 was incubated with CY3-labeled SU056@GelMA in a shaker at 37 °C with a speed of 100 rpm. At predetermined time points, the supernatant was completely collected and replaced with fresh solution. The release of CY3-labeled SU056 was then measured using a multimode reader.

CY3-labeled SU056 was either directly injected or incorporated into GelMA prior to injection into rat intervertebral discs. At designated time points (0, 3, 7, and 21 days), in vivo imaging was used to capture fluorescent dye retention within the rat intervertebral discs.

### Biosafety evaluation of SU056@GelMA

To evaluate the long-term in vivo biosafety of SU056@GelMA after intradiscal implantation, 6-week-old male rats were assigned to PBS, GelMA, or SU056@GelMA injection groups (*n *= 5 per group). Caudal intervertebral disc injection was performed using the same puncture and microinjection procedures described above. At 8 weeks after implantation, rats were euthanized, and peripheral blood, major organs (heart, liver, spleen, lung, and kidney), and the operated caudal disc segments were collected for systemic and local safety assessments.

Complete blood count analysis was performed to assess white blood cells, red blood cells, platelets, and hemoglobin. Serum biochemical indices, including alanine aminotransferase, aspartate aminotransferase, blood urea nitrogen, and creatinine, were measured to evaluate liver and renal function. Major organs were fixed, embedded, sectioned, and stained with HE. The caudal disc injection site was processed for Masson's trichrome staining to evaluate collagen deposition and fibrosis, and IF staining was performed for α-smooth muscle actin (α-SMA), interleukin-1β (IL-1β), interleukin-6 (IL-6), CD68/CD80, and CD68/CD206. CD68/CD80 and CD68/CD206 were used to evaluate M1-like and M2-like macrophage-associated infiltration, respectively. Image acquisition and quantitative analysis were performed according to the protocols described above.

### Statistics

Statistical analyses were performed using GraphPad Prism (version 9). Data are presented as the mean ± s.e.m. Comparisons between two groups were performed using an unpaired *t*-test. Correlation analysis was conducted using simple linear regression to assess the linear relationship between variables. Statistical significance was set at *P *< 0.05. Significance levels were indicated as follows: *P < 0.05 (statistically significant), **P < 0.01 (significantly different), and ***P < 0.001 (highly significant).

## Results

### HDAC1 expression progressively decreases during IVDD progression

**Fig. 1:**
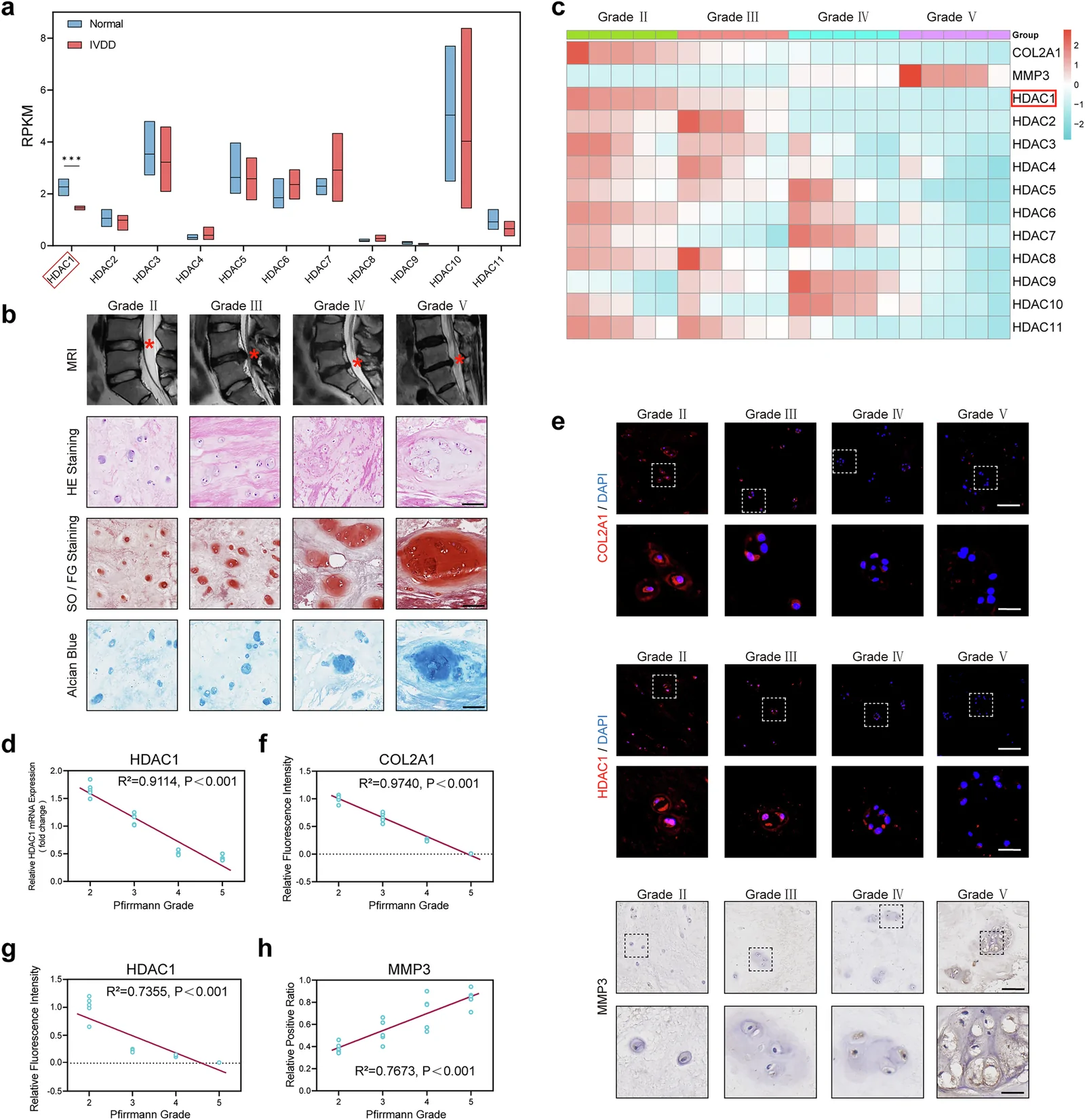
HDAC expression and correlation with intervertebral disc degeneration (IVDD). **a** RNA-seq analysis of *HDACs* expression in normal and IVDD tissues, stratified into Normal and IVDD groups (*n *= 5). **b** Representative magnetic resonance images of human nucleus pulposus (NP) tissues, accompanied by hematoxylin and eosin (HE), safranin O and fast green (SO & FG), and Alcian Blue staining. Scale bar: 100 μm. **c** RT-qPCR generated heatmap revealing transcriptional levels of *COL2A1*, *MMP3*, and *HDACs* across different Pfirrmann degeneration grades (grades II–V, *n *= 5 per group). **d** Correlation analysis between *HDAC1* transcription levels and Pfirrmann degenerative grades (grades II–V, *n *= 5 per group). **e–h** IF and IHC staining of COL2A1, HDAC1, and MMP3 in NP tissues, with correlation analysis between protein expression and Pfirrmann degenerative grades (grades II–V, *n *= 5 per group). Scale bars: low magnification 100 μm; high magnification 25 μm. Correlation analyses performed using simple linear regression (panels d, f, g, and h).

To investigate the expression dynamics of HDACs in IVDD, we used the Mass Spectrometry Database (MSDB) bioinformatics tool developed by Tian et al.^32^. Leveraging RNA-seq data (Supplementary Fig. 1a), we conducted a comparative analysis of gene expression profiles between normal and degenerated NP tissues^33–36^. Our findings revealed a statistically significant downregulation of *HDAC1* in degenerative NP tissues. Although other *HDACs* showed marginal variations in expression, these alterations were not statistically significant (Fig. 1a and Supplementary Fig. 1b, c).

Our initial observations were systematically validated by collecting and analyzing 35 NP tissue samples from surgical patients (Supplementary Table 1). Using the established Pfirrmann grading system^21^, we stratified the patients into four distinct cohorts: grade II (*n* = 7, mean age 51 years), grade III (*n *= 10, mean age 49 years), grade IV (*n *= 13, mean age 58 years), and grade V (*n *= 5, mean age 57 years). A progressive decline in MRI T2-weighted disc signal intensity, accompanied by increasing disc collapse, was meticulously documented as the degeneration grades advanced. Comprehensive histological examinations included HE, SO & FG, and Alcian Blue staining. Degenerated NP tissues manifest characteristic pathological features, including progressive matrix disintegration, focal calcification, and abnormal NPC clustering with chondroid metaplasia. These degenerative changes were most pronounced in grade IV and V tissue specimens (Fig. 1b).

NPCs were carefully extracted from selected grade II–V samples, cultured to passage 1, and subjected to RNA extraction for RT-qPCR. The results demonstrated a nuanced molecular progression: transcriptional levels of the ECM synthesis enzyme collagen type 2 alpha 1 chain (*COL2A1*) exhibited a significant inverse relationship with IVDD grades, whereas transcription of the degradation enzyme matrix metalloproteinase 3 (*MMP3*) correspondingly increased. Concurrently, *HDAC1* transcript levels showed a parallel decline with advancing disc degeneration (Fig. 1c).

Rigorous statistical analysis employing simple linear regression revealed compelling correlations between gene transcription levels and Pfirrmann grades. *HDAC1* and *COL2A1* displayed statistically significant negative linear correlations with the severity of degeneration, whereas *MMP3* exhibited a robust positive linear relationship (Fig. 1d and Supplementary Fig. 1c).

To further substantiate these molecular observations, we conducted comprehensive IF and IHC analyses of the patient tissue sections (Fig. 1e). Protein-level investigations corroborated our transcriptional findings; COL2A1 expression in NPCs demonstrated a statistically significant negative linear correlation with Pfirrmann grades (Fig. 1f), whereas MMP3 expression revealed a corresponding positive linear correlation (Fig. 1h). Notably, HDAC1 protein expression in NPCs was inversely correlated with the degeneration grade (Fig. 1g).

These multifaceted analyses provide compelling evidence that HDAC1 expression in NPCs undergoes a progressive and notable reduction during IVDD progression. Our findings elucidate the molecular mechanisms underlying disc degeneration and suggest that aberrant HDAC1 expression represents a critical pathogenic factor in this complex degenerative process.

### HDAC1 maintains NPC metabolic homeostasis

**Fig. 2:**
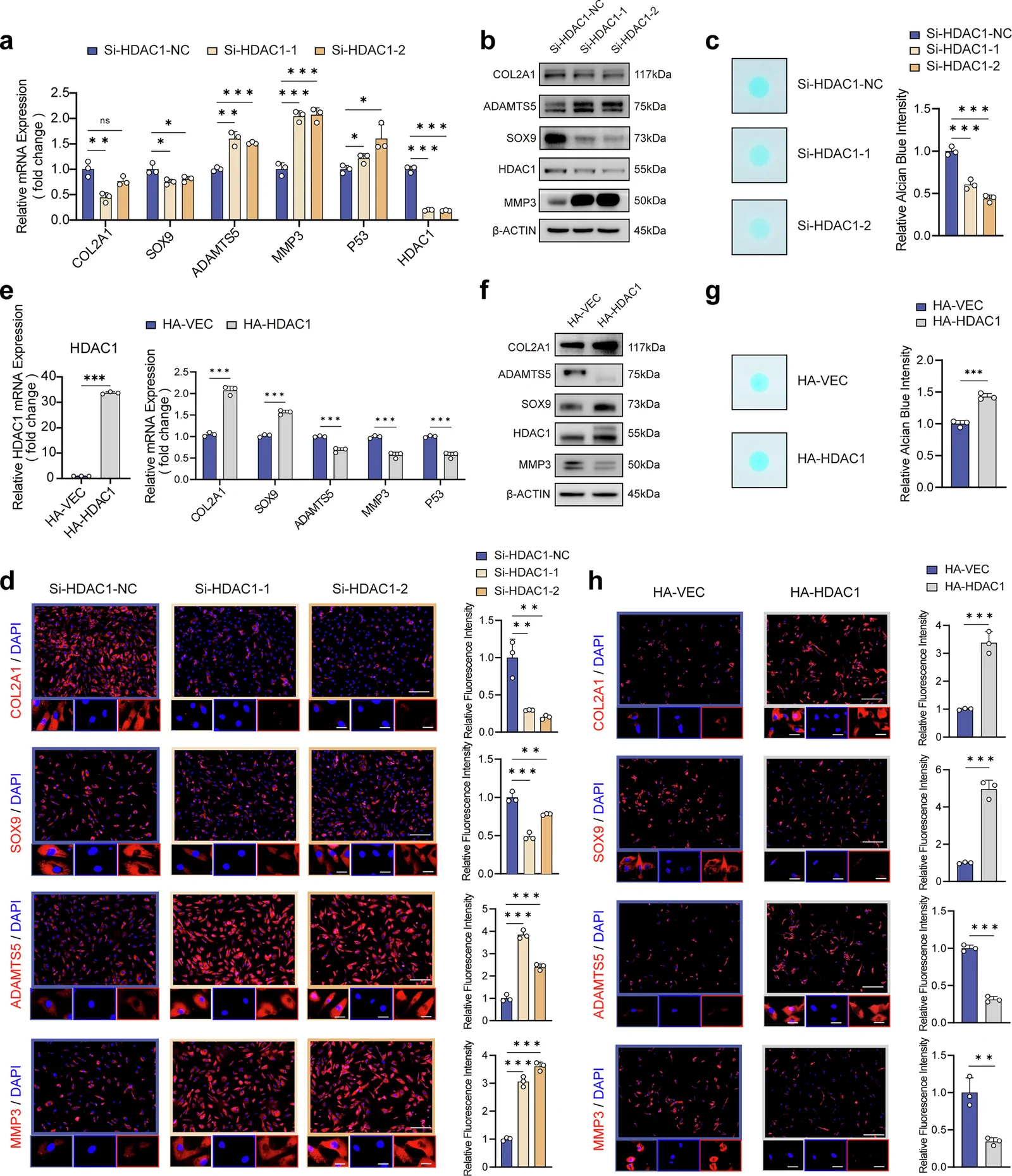
HDAC1 regulation of the extracellular matrix (ECM) in nucleus pulposus cells (NPCs). **a** RT-qPCR analysis of *COL2A1*, *SOX9*, *ADAMTS5*, and *MMP3* transcriptional levels after *HDAC1* knockdown (*n *= 3 biological replicates). **b** Representative western blot images showing protein levels of COL2A1, ADAMTS5, SOX9, HDAC1, and MMP3 before and after HDAC1 knockdown (*n *= 3 biological replicates). **c** High-density culture and Alcian Blue staining of human NPCs before and after HDAC1 knockdown, with quantitative intensity analysis (*n *= 3 biological replicates). **d** IF images of COL2A1, SOX9, ADAMTS5, and MMP3 in human NPCs before and after HDAC1 knockdown, with quantification. Scale bars: low magnification 200 μm; high magnification 50 μm. **e** RT-qPCR results showing transcriptional levels of *COL2A1*, *SOX9*, *ADAMTS5*, *MMP3*, *TP53*, and HDAC1 after HDAC1 overexpression (*n *= 3 biological replicates). **f** Representative western blot images demonstrating protein expression levels of COL2A1, ADAMTS5, SOX9, HDAC1, and MMP3 (*n *= 3 biological replicates). **g** High-density culture and Alcian Blue staining of human NPCs before and after HDAC1 overexpression, with quantitative analysis (*n* = 3 biological replicates). **h** IF staining of COL2A1, SOX9, ADAMTS5, and MMP3 in human NPCs. Scale bars: low magnification 200 μm; high magnification 50 μm. Data presented as mean ± s.e.m. Statistical differences calculated using unpaired *t*-test, where *P < 0.05, **P < 0.01, ***P < 0.001 (panels a, c, d, e, g, and h).

We implemented a targeted siRNA knockdown approach to elucidate the functional role of HDAC1 in NPCs. RT-qPCR revealed a marked reduction in *HDAC1* expression after si-HDAC1 transfection. This molecular intervention triggered a cascade of cellular responses; ECM synthesis enzymes *COL2A1* and SRY-box transcription factor 9 (*SOX9*) were significantly downregulated, whereas ECM degradation enzymes a disintegrin and metalloproteinase with thrombospondin motifs 5 (*ADAMTS5*) and *MMP3*, in addition to the senescence marker *TP53*, were markedly upregulated (Fig. 2a). Complementary protein-level investigations via western blotting corroborated these transcriptional observations, confirming the molecular perturbations induced by HDAC1 silencing (Fig. 2b). Staining for the senescence marker β-galactosidase (β-gal) provided additional evidence of accelerated cellular aging, consistent with the observed *TP53* upregulation (Supplementary Fig. 2a).

High-density culture experiments and Alcian Blue staining offer a morphological perspective on ECM synthesis^37^. Glycomatrix secretion, as visualized by the Alcian Blue staining intensity and patch characteristics, demonstrated a significant reduction in ECM synthesis after *HDAC1* knockdown (Fig. 2c). This macroscopic observation was further substantiated by IF analyses, which revealed the decreased expression of the ECM synthesis enzymes COL2A1 and SOX9, accompanied by increased expression of ADAMTS5 and MMP3 (Fig. 2d).

We engineered exogenous HDAC1 overexpression in NPCs to investigate this inverse scenario using a lentiviral HA–HDAC1 plasmid delivery system. Comprehensive molecular characterization using RT-qPCR and western blotting confirmed the substantial HDAC1 upregulation. This intervention precipitated remarkable molecular recalibration; NPC matrix synthesis markers *COL2A1* and *SOX9* exhibited significant upregulation, whereas degradation markers *ADAMTS5* and *MMP3* demonstrated pronounced downregulation. Concurrently, *TP53* transcriptional expression was notably decreased (Fig. 2e, f).

High-density culture experiments, visualized through enhanced Alcian Blue staining, revealed increased matrix staining intensity, indicating restored and enhanced ECM synthesis (Fig. 2g). IF analysis further corroborated these findings, demonstrating increased COL2A1 and SOX9 expression, with decreased ADAMTS5 and MMP3 expression following HDAC1 overexpression (Fig. 2h).

These comprehensive investigations spanning the transcriptional, translational, and morphological domains provide compelling evidence for the pivotal role of HDAC1 in maintaining NPC metabolic homeostasis. When HDAC1 expression is compromised, NPCs shift toward a degenerative phenotype characterized by suppressed ECM synthesis, accelerated ECM degradation, and heightened senescence. Conversely, elevated HDAC1 expression effectively counteracted these deleterious cellular transformations, underscoring its critical function in preserving the cellular metabolic equilibrium and potentially mitigating IVDD progression.

### HDAC1 mediates YBX1 deacetylation and ubiquitination-dependent degradation

**Fig. 3:**
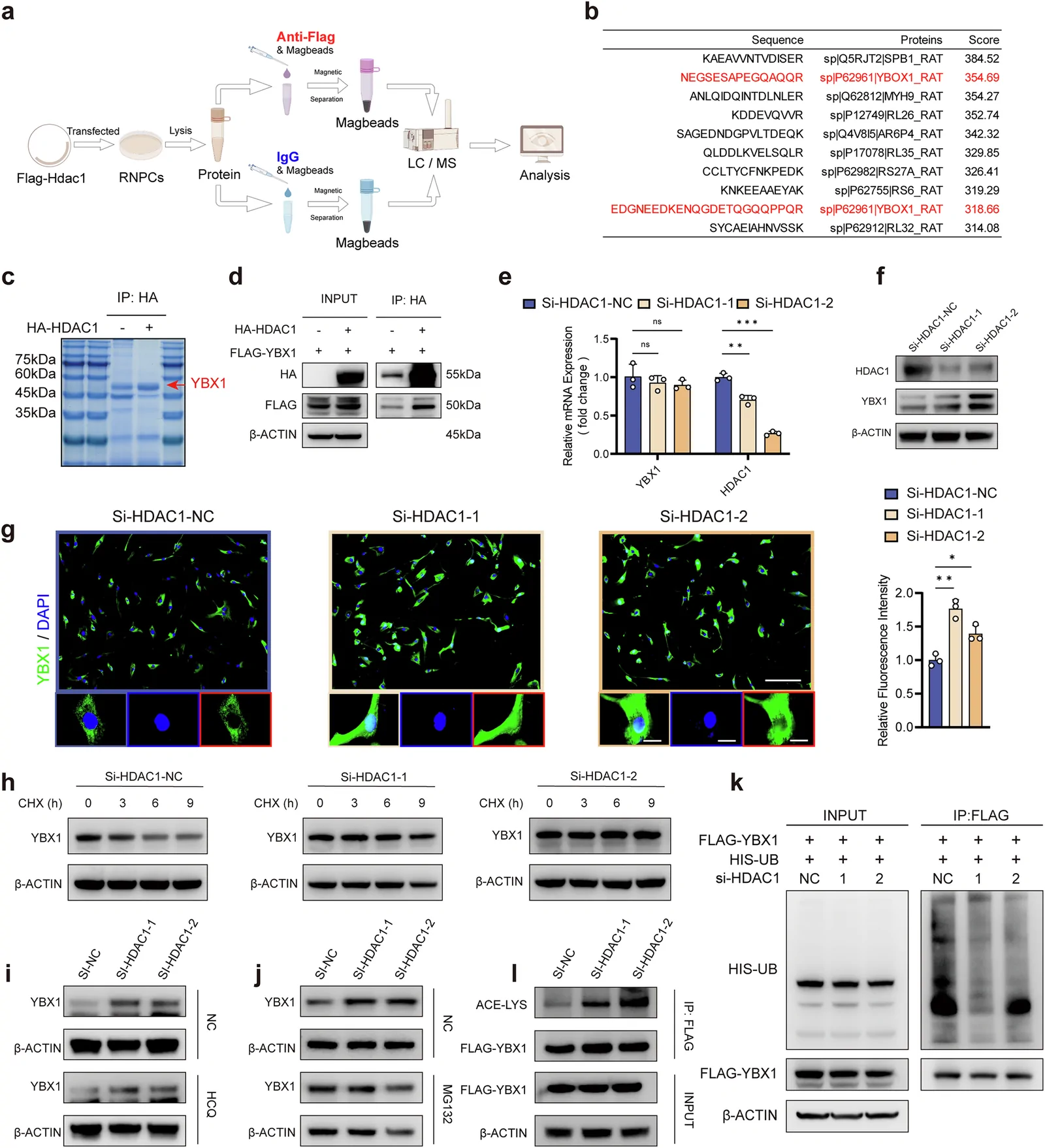
HDAC1–YBX1 interaction characterization. **a** Protein mass spectrometry (MS) preparation and analysis workflow. **b** Top ten peptide segments from MS results and corresponding proteins. **c** Exogenous HA–HDAC1 expression in HEK293T cells, followed by HA antibody immunoprecipitation (IP) and Coomassie blue staining. **d** Verification of HDAC1–YBX1 binding through exogenous expression of HA–HDAC1 and FLAG–YBX1 in HEK293T cells. **e** RT-qPCR results showing *YBX1* transcriptional changes after *HDAC1* silencing (*n *= 3 biological replicates). **f** Representative western blot images demonstrating YBX1 protein-level changes after HDAC1 silencing (*n *= 3 biological replicates). **g** IF staining of YBX1 in human NPCs. Scale bars: low magnification 200 μm; high magnification 50 μm. **h** Western blot images showing YBX1 protein degradation after treatment with cycloheximide at 0, 3, 6, and 9 h post-si-HDAC1 transfection (*n* = 3 biological replicates). **i, j** Western blot images demonstrating YBX1 protein levels after hydroxychloroquine or MG132 treatment 24 h post-si-HDAC1 transfection (*n *= 3 biological replicates). **k** IP with FLAG antibody after FLAG–YBX1 and HIS–UB exogenous expression and HDAC1 silencing in HEK293T cells (*n *= 3 biological replicates). **l** FLAG–YBX1 expressed in HEK293T cells with FLAG IP. Representative western blots of acetylated lysine (*n *= 3 biological replicates). Data presented as mean ± s.e.m. Statistical differences calculated using unpaired *t*-test, where *P < 0.05, **P < 0.01, ***P < 0.001 (panels e and g).

Leveraging the inherent deacetylase functionality of HDAC1, we conducted a comprehensive MS analysis to identify its protein binding partners. Given the challenging proliferation of human NPCs, which precludes large-scale protein enrichment, we developed a FLAG–Hdac1 plasmid based on the rat genome. Following lentiviral transfection to achieve Hdac1 overexpression in rat NPCs, we processed the cellular lysates using magnetic bead-conjugated antibodies and employed a rigorous liquid chromatography–MS detection and database interrogation protocol (Fig. 3a, created by Figdraw).

Interestingly, proteomic analysis revealed two peptide segments corresponding to the Ybx1 protein among the top ten binding candidates (Fig. 3b). A comprehensive MS review revealed 15 distinct Hdac1-binding Ybx1 peptide segments (Supplementary Fig. 3a). This finding prompted further investigation, particularly given the established reputation of YBX1 as a nucleic acid-binding protein with critical roles in transcriptional and translational regulation.

We hypothesized that HDAC1, which functions beyond its canonical histone modification role, modulates YBX1 through acetylation-mediated mechanisms in NPCs. A series of validation experiments using HEK293T cells supported this hypothesis. Exogenous HA–HDAC1 expression, followed by IP, revealed a distinct YBX1-associated protein band (Fig. 3c). Subsequent coexpression of HA–HDAC1 and FLAG–YBX1 conclusively demonstrated direct molecular interactions through co-IP (Fig. 3d). Mechanistic investigations using gene knockdown experiments provided insights into their molecular relationships. RT-qPCR analysis revealed no substantial transcriptional alterations in *YBX1* upon *HDAC1* silencing (Fig. 3e). Conversely, western blotting and IF experiments demonstrated pronounced protein-level changes; *HDAC1* knockdown triggered marked YBX1 accumulation, particularly in nuclear and cytoplasmic compartments (Fig. 3f, g).

We used pharmacological interventions to elucidate the precise degradation mechanism. Treatment with cycloheximide, a protein synthesis inhibitor, revealed that *HDAC1* silencing notable impaired YBX1 degradation kinetics (Fig. 3h), indicating the critical role of HDAC1 in YBX1 turnover^38^. Mechanistic pathway investigations using the autophagy inhibitor hydroxychloroquine and proteasome inhibitor MG132 have provided definitive insights^39^. Western blotting demonstrated that, although blocking the autophagy pathway did not prevent YBX1 accumulation upon *HDAC1* silencing, proteasome inhibition completely abrogated this effect (Fig. 3i, j). This establishes that the proteasomal pathway is the primary mechanism of HDAC1-mediated YBX1 degradation.

Complementary experiments in HEK293T cells involving the exogenous expression of FLAG–YBX1 and HIS–UB corroborated these findings. *HDAC1* silencing markedly reduced YBX1 ubiquitination, providing molecular evidence of the role of HDAC1 in facilitating protein degradation (Fig. 3k). Western blotting provided definitive evidence of HDAC1 deacetylation, demonstrating a marked reduction in YBX1 acetylation levels (Fig. 3l).

In summary, our comprehensive investigation reveals the molecular mechanism by which HDAC1 orchestrates YBX1 protein homeostasis. Through concurrent deacetylation and ubiquitination, HDAC1 effectively targets YBX1 for proteasomal degradation, representing a post-translational regulatory mechanism with profound implications for cellular protein stability control.

### YBX1 drives ECM degradation in NPCs

**Fig. 4:**
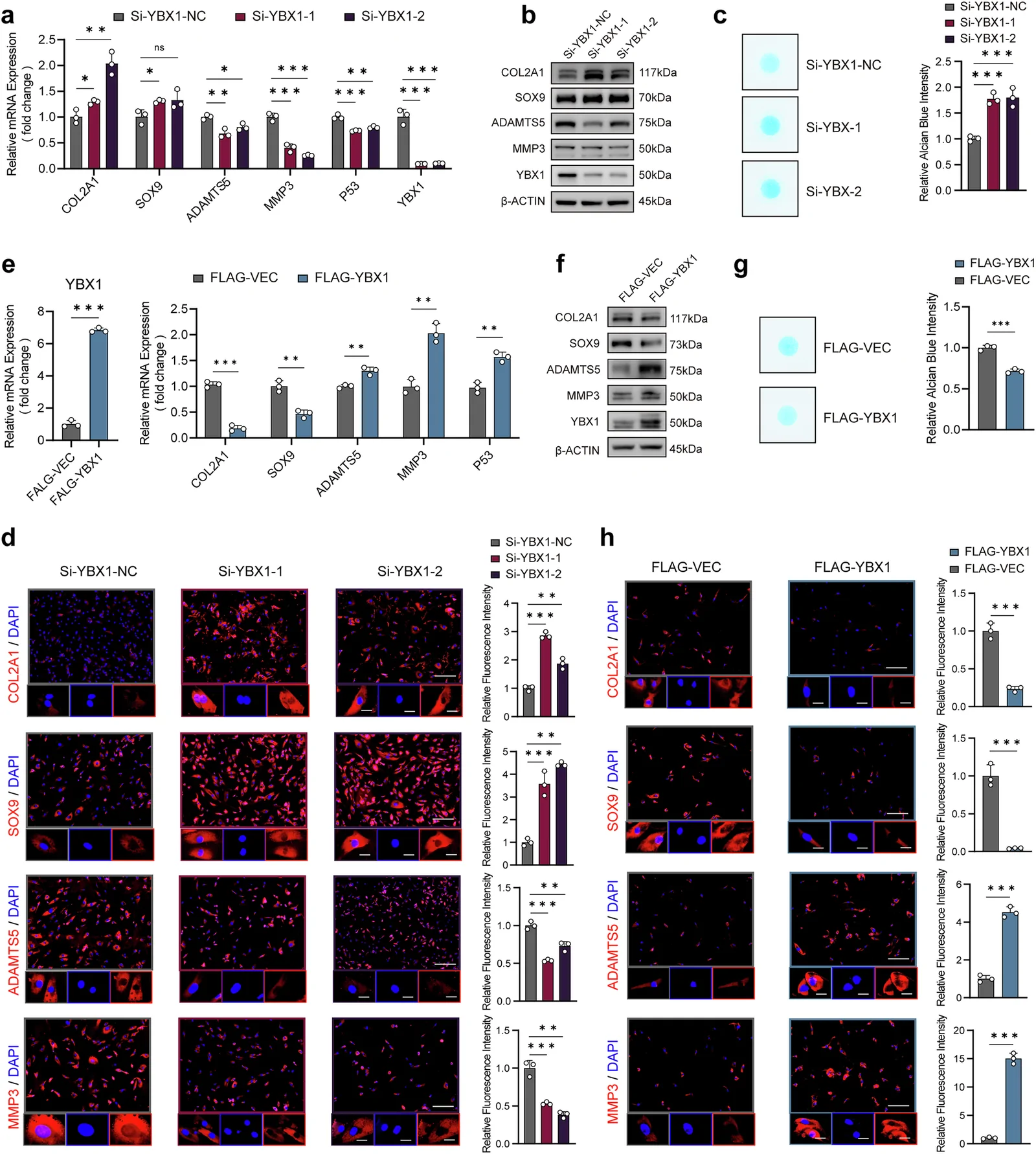
YBX1 modulation of nucleus pulposus cell (NPC) metabolism. **a** RT-qPCR results showing changes in extracellular matrix (ECM) metabolism markers (*COL2A1*, *SOX9*, *ADAMTS5*, *MMP3*) and senescence indicator *TP53* after YBX1 silencing (*n* = 3 biological replicates). **b** Representative western blot images demonstrating protein-level changes in ECM metabolism markers COL2A1, SOX9, ADAMTS5, and MMP3 after YBX1 silencing (*n* = 3 biological replicates). **c** High-density culture and Alcian Blue staining of NPCs after YBX1 silencing, with quantitative staining intensity analysis (*n *= 3 biological replicates). **d** IF images and quantitative fluorescence intensity analysis showing COL2A1, SOX9, ADAMTS5, and MMP3 expression changes after YBX1 silencing (*n *= 3 biological replicates). **e** RT-qPCR results revealing transcriptional changes in ECM metabolism markers (*COL2A1*, *SOX9*, *ADAMTS5*, *MMP3*) and senescence indicator *TP53* after YBX1 overexpression (*n* = 3 biological replicates). **f** Representative western blot images showing protein-level changes in ECM metabolism markers COL2A1, SOX9, ADAMTS5, and MMP3 after YBX1 overexpression (*n* = 3 biological replicates). **g** High-density culture and Alcian Blue staining of NPCs after YBX1 overexpression, with quantitative staining intensity analysis (*n *= 3 biological replicates). **h** IF images demonstrating COL2A1, SOX9, ADAMTS5, and MMP3 expression changes after YBX1 overexpression (*n *= 3 biological replicates). Data presented as mean ± s.e.m. Statistical differences calculated using unpaired *t*-test, where *P < 0.05, **P < 0.01, ***P < 0.001 (panels a, c, d, e, g, and h).

To explore the functional role of YBX1 in NPCs, we performed a comprehensive molecular investigation using gene silencing and overexpression strategies. We used siRNA to knockdown YBX1 to uncover the complex cellular response. RT-qPCR analysis demonstrated significant YBX1 suppression accompanied by remarkable molecular recalibration. Notably, we observed pronounced upregulation of the ECM synthesis enzymes *COL2A1* and *SOX9*, concurrent with downregulation of the degradation enzymes *ADAMTS5* and *MMP3*, and reduced expression of the senescence marker *TP53* (Fig. 4a). Protein-level investigations via western blotting comprehensively corroborated these transcriptional alterations, confirming decreased YBX1 expression and the corresponding shifts in key metabolic markers (Fig. 4b). Supplementary cellular senescence assessment through β-gal staining provided morphological evidence of reduced cellular aging following YBX1 knockdown (Supplementary Fig. 2b). High-density culture experiments, visualized by Alcian Blue staining, further substantiated these findings. The enhanced matrix staining intensity following YBX1 silencing unequivocally demonstrated improved ECM synthesis (Fig. 4c). IF analysis provided insights into the molecular mechanisms involved, revealing a significant reconfiguration of cellular protein expression. YBX1 knockdown increased the expression of the ECM synthesis enzymes COL2A1 and SOX9, accompanied by suppression of the degradation enzymes ADAMTS5 and MMP3 (Fig. 4d).

We subsequently investigated the effects of YBX1 overexpression via lentiviral-mediated FLAG–YBX1 exogenous expression. The molecular landscape underwent a dramatic transformation; RT-qPCR and western blotting consistently demonstrated *YBX1* upregulation, coinciding with decreased ECM synthesis enzyme expression and increased degradation enzyme and senescence marker levels (Fig. 4e–g). IF investigations provided definitive visual confirmation of these molecular alterations. Increased YBX1 expression corresponded to profound changes in cellular protein expression, including a pronounced reduction in COL2A1 and SOX9, coupled with a significant enhancement of ADAMTS5 and MMP3 expression (Fig. 4h).

We observed that with the increase in degeneration grade, the expression of YBX1 in NP tissue samples exhibited a gradual upward trend (Supplementary Fig. 3b). These meticulously designed experiments employing both gain- and loss-of-function approaches provided compelling and multilayered evidence for the critical role of YBX1 in NPC pathophysiology. This bidirectional experimental strategy demonstrated that YBX1 serves as a pivotal molecular driver of ECM degradation, cellular senescence, and metabolic dysregulation in NPCs.

### YBX1 modulates inflammatory chemokine expression in NPCs

**Fig. 5:**
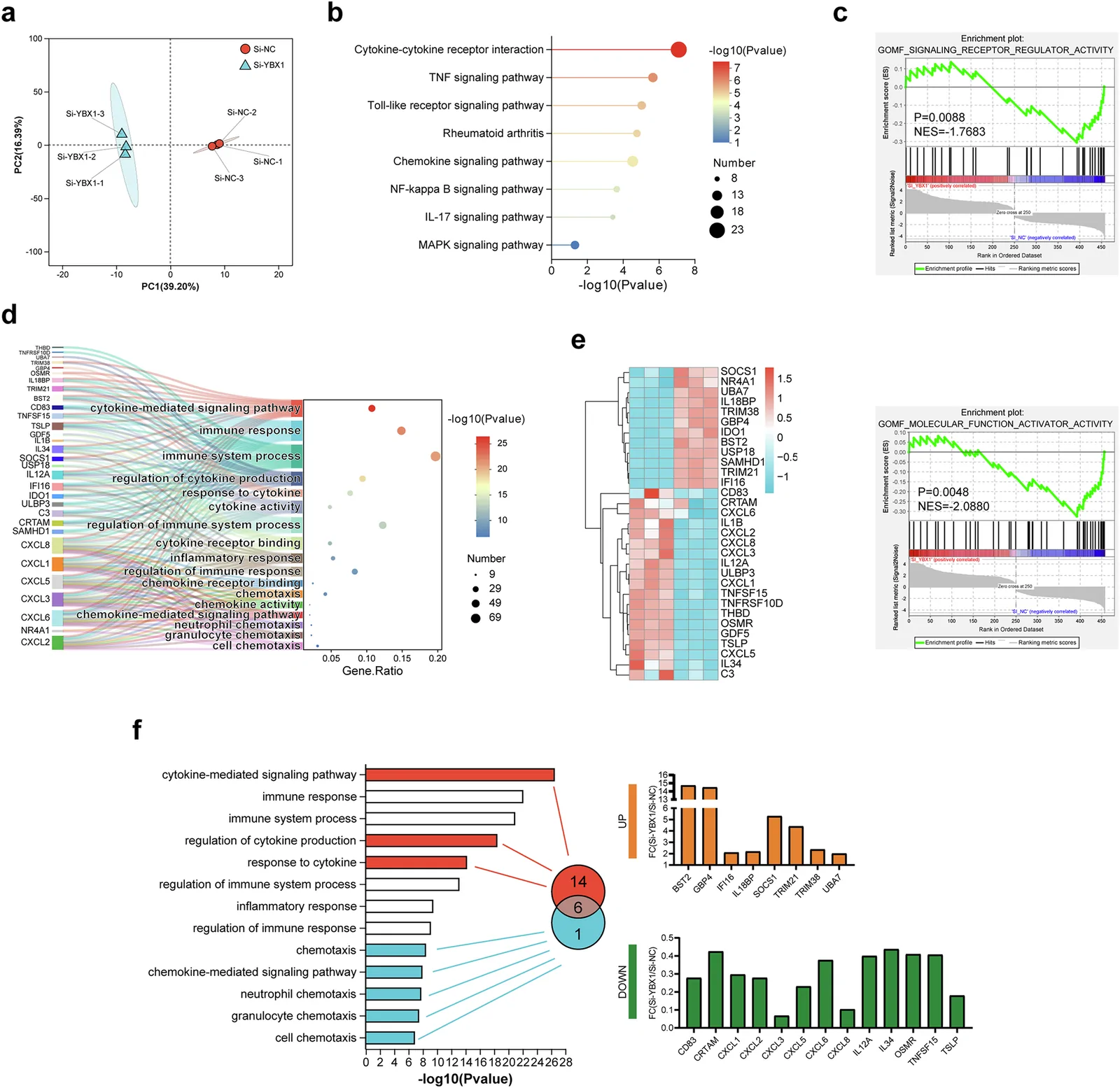
Transcriptomic analysis of YBX1 knockdown effects. **a** Principal component analysis (PCA) showing sample clustering for si-NC and si-YBX1 groups (*n *= 3 biological replicates). **b** Kyoto Encyclopedia of Genes and Genomes enrichment analysis revealing pathways enriched by differentially expressed genes after YBX1 silencing. **c** Gene set enrichment analysis showing gene expression changes in signal receptor regulatory activity and molecular function activator pathways before and after YBX1 silencing. **d** Sankey bubble plot based on Gene Ontology (GO) enrichment analysis, displaying major gene expression changes and their associated pathways after YBX1 silencing. **e** Heatmap illustrating expression changes of key genes in GO-enriched pathways after YBX1 silencing (*n *= 3 biological replicates). **f** Overlap and expression changes of genes in cytokine and chemokine pathways.

Delineating the molecular regulatory mechanisms of YBX1 in NPCs, we performed RNA-seq after targeted gene silencing. Prinicpal component analyses revealed concentrated clustering for both si-normal control (NC) and si-*YBX1* groups, demonstrating robust intragroup sample consistency and experimental reproducibility (Fig. 5a). Kyoto Encyclopedia of Genes and Genomes enrichment analysis unveiled that differentially expressed genes were predominantly clustered in critical inflammatory pathways, including cytokine–receptor interactions, tumor necrosis factor signaling, and Toll-like receptor signaling cascades (Fig. 5b). Gene set enrichment analysis revealed pronounced transcriptional alterations in the signal receptor regulatory activity and molecular function activator pathways upon *YBX1* silencing (Fig. 5c). This suggests that YBX1 plays a role in regulating intercellular communication and interactions, potentially through modulating signaling pathways involved in cell–cell crosstalk.

A comprehensive Sankey bubble plot generated through Gene Ontology enrichment analysis highlighted the key molecular players: *SOCS1*, *NR4A1*, *UBA7*, *C3*, *IL34*, and *CXCL5*. These genes were predominantly enriched in cell chemotaxis and cytokine-related molecular circuits (Fig. 5d). A meticulously constructed heatmap provided an intuitive visualization of the gene expression dynamics (Fig. 5e). By leveraging substantial gene enrichment in the cytokine and chemokine pathways, we conducted a granular analysis of the upregulated and downregulated genes. A Venn diagram analysis revealed six genes that intersected with *CXCL1–3*, *CXCL5*, *CXCL6*, and *CXCL8*. Notably, these chemokines exhibited synchronous and significant downregulation after *YBX1* knockdown (Fig. 5f), with all identified chemokines demonstrating potent neutrophil chemotactic capabilities^40–42^. We analyzed the expression of CXCL8 in human NP tissue and observed a progressive increase in its expression levels with advancing Pfirrmann grade (Supplementary Fig. 3c).

The traditional view holds that the intervertebral disc is an immune-privileged organ^43^; however, recent single-cell sequencing has shown that IVDD features infiltration of inflammatory cells, macrophages, T cells, and neutrophils, which release inflammatory cytokines that promote local pain and enhance senescence in NPCs^44–46^. Therefore, we propose that YBX1 modulates the ECM metabolism of NPCs and plays a pivotal role in shaping the inflammatory microenvironment during NPC degeneration. Notably, *HDAC1* expression is downregulated in degenerative NPCs, leading to intracellular accumulation of YBX1. This accumulation promotes neutrophil chemokine secretion, drives neutrophil infiltration, and triggers an inflammatory cascade that ultimately exacerbates NPC degeneration.

### HDAC1 deficiency exacerbates IVDD through YBX1 accumulation

**Fig. 6:**
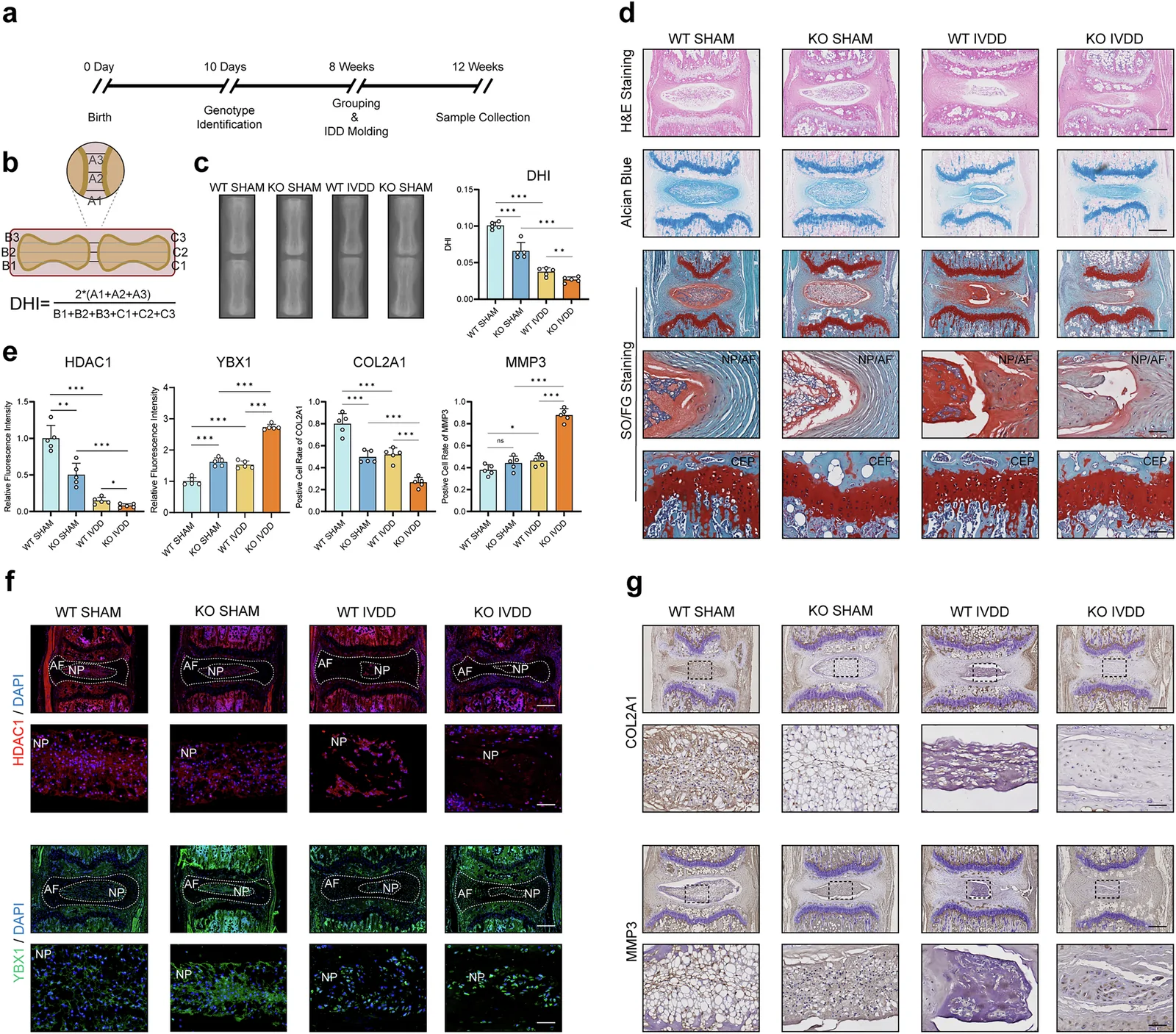
Hdac1 knockout (KO) mouse model characterization. **a** Animal experiment timeline for gene KO mouse model. **b** Schematic of the disc height index (DHI) calculation method for mouse tail specimens. **c** Representative X-ray images of mouse tail specimens and DHI quantitative analysis (*n* = 5 biological replicates). **d** Histological staining (HE, SO & FG, and Alcian Blue) of intervertebral disc sections from different groups. Magnified views of the NP–annulus fibrosus (AF) junction and cartilage endplate. Scale bars: low magnification 300 μm; high magnification 60 μm. **e** Quantitative analysis of IF and IHC of Hdac1, Ybx1, Col2a1, and Mmp3 (*n *= 5 biological replicates). **f** IF images showing Hdac1 and Ybx1 expression in NP after Hdac1 KO and disc puncture. Scale bars: low magnification 300 μm; high magnification 60 μm. **g** Representative IHC images demonstrating Mmp3 expression levels in NPCs. Scale bars: low magnification 300 μm; high magnification 60 μm. Data presented as mean ± s.e.m. Statistical differences calculated using unpaired *t*-test, where *P < 0.05, **P < 0.01, ***P < 0.001 (panels c, e, and f).

Extending our previous investigations into the mechanistic role of HDAC1 in NPCs, we systematically explored the effect of Hdac1 downregulation on mouse tail IVDD. We successfully generated *Hdac1* gene knockout mice (model schematic in Supplementary Fig. 4a) and found that complete *Hdac1* knockout resulted in embryonic lethality. Consequently, we stratified mice with partial *Hdac1* deletion (KO) and those with minimal genetic modification (WT), and demonstrated through western blotting that the expression of *Hdac1* in NP tissue was significantly decreased (Supplementary Fig. 4d). After comprehensive genetic characterization (Supplementary Fig. 4b, c), we divided the mice into experimental groups: WT SHAM, WT IVDD, KO SHAM, and KO IVDD (*n *= 5 per group). Comprehensive sample analysis incorporated X-ray imaging, multimodal staining techniques, IF, and IHC (Fig. 6a).

Fluorescence intensity analysis revealed significantly diminished Hdac1 signals in KO SHAM mice compared with WT SHAM mice, with similar trends observed in IVDD groups. Notably, IVDD samples consistently demonstrated reduced Hdac1 fluorescence across genotypes, corroborating the findings from human NPC specimens (Fig. 6f). X-ray-based disc height index measurements (Fig. 6b) confirmed progressive disc degeneration. Comparative analyses demonstrated significant disc height reductions in IVDD groups relative to SHAM controls, with KO groups exhibiting more pronounced deterioration than their WT counterparts (Fig. 6c), underscoring the pivotal role of HDAC1 in maintaining disc structural integrity. Histological examinations using HE, SO & FG, and Alcian Blue staining revealed nuanced morphological transformations. The WT SHAM groups presented a typical disc morphology, whereas the KO SHAM samples displayed reduced glycomatrix accumulation. Progressive degeneration in the WT IVDD group manifested as disc collapse, NPC atrophy, cellular clustering, and annulus fibrosus (AF) disruption. The KO IVDD group exhibited more severe pathological changes characterized by extreme disc collapse and near-complete structural disintegration (Fig. 6d). Targeted Ybx1 IF revealed markedly elevated fluorescence intensity in the KO groups, reaffirming the critical role of HDAC1 in YBX1 protein degradation. Consistently, IVDD samples demonstrated a progressive increase in Ybx1 expression under all experimental conditions (Fig. 6e, f). Notably, Cxcl8 demonstrated a pattern similar to that of Ybx1 (Supplementary Fig. 6a), further substantiating the findings from the RNA-seq analysis. Hdac1 deficiency also intensified CD68/CD80-positive M1-like macrophage infiltration and MPO/ELA2-positive neutrophil infiltration in degenerative mouse NP tissue (Supplementary Fig. 7a). IHC analyses provided molecular-level insights; KO groups exhibited significantly reduced Col2a1 expression concurrent with elevated Mmp3 levels compared with WT controls (Fig. 6e, g). The IVDD group showed further amplification of these molecular alterations, with pronounced downregulation of ECM proteins and upregulation of protein degradation.

Collectively, these findings elucidate the critical mechanism by which HDAC1 deficiency promotes YBX1 accumulation in NPCs. This molecular perturbation triggers a cascading effect, including decreased ECM protein synthesis, increased degradation marker expression, enhanced chemokine-associated inflammatory cell infiltration, and accelerated progression of IVDD.

### Preparation, characterization, and biocompatibility evaluation of SU056@GelMA

To target YBX1 and mitigate IVDD, we used SU056, a selective YBX1 inhibitor, and performed comprehensive dosage optimization using concentration gradient and CCK8 viability assays. Rigorous screening identified 1.25 μM as the optimal in vitro concentration for rat cells (Supplementary Fig. 5a–c), demonstrating a capacity to partially mitigate *Hdac1* knockdown-induced cellular perturbations (Supplementary Fig. 5d). IVDD damages the AF and causes NPC leakage. Direct drug injection may exacerbate this damage, leading to leakage. GelMA is a biocompatible hydrogel that maintains the mechanical stability of the disc and seals the AF, thereby preventing NPC and drug leakage. Integration of SU056 into 10% GelMA yielded a mixed hydrogel with a 5 μM final concentration, which maintained NPC viability in vitro (Supplementary Fig. 5e).

**Fig. 7:**
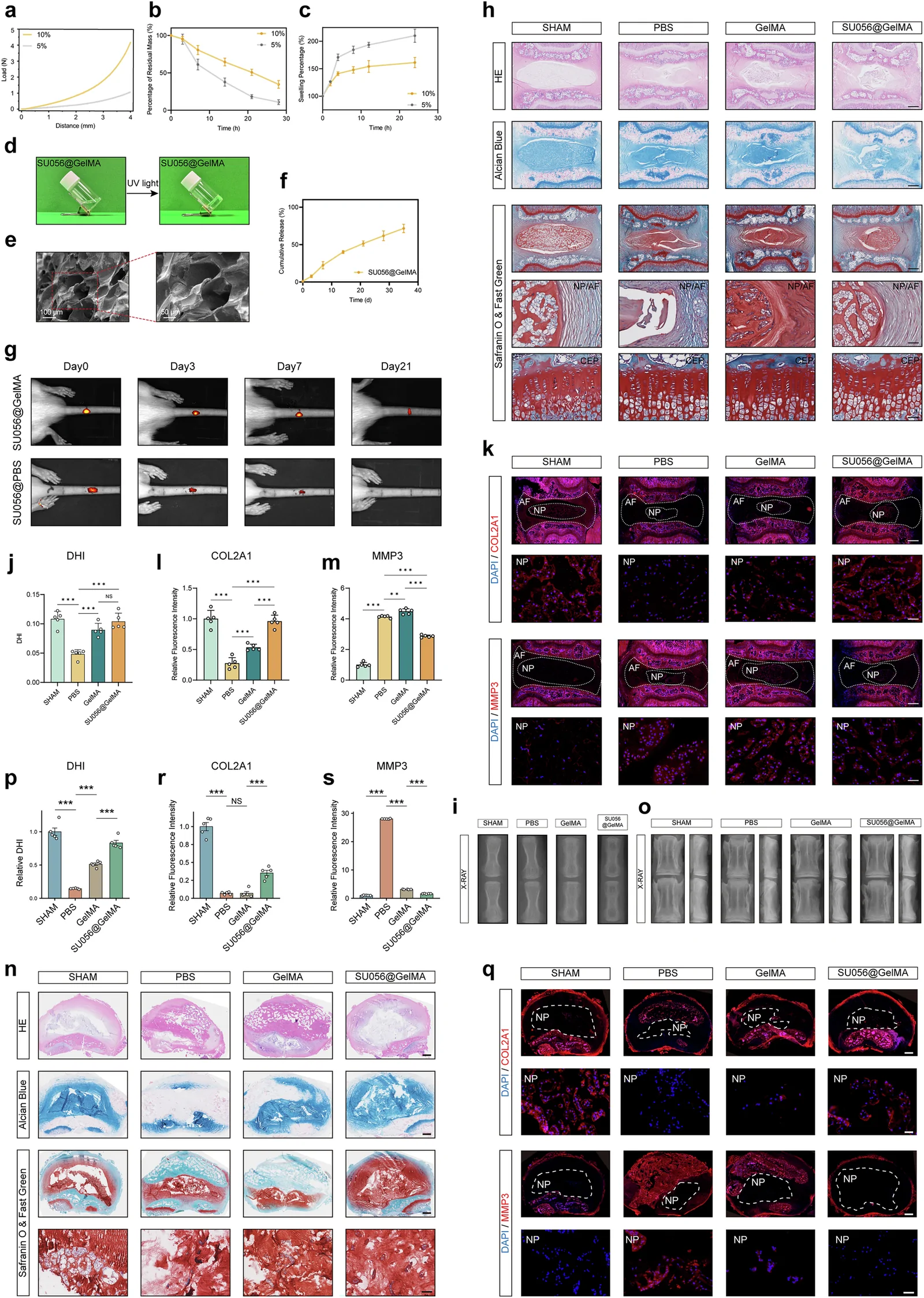
SU056@GelMA hydrogel characterization and therapeutic intervention in rat caudal and New Zealand White rabbit lumbar IVDD models. **a–c** Stress–strain curves, degradation rate, and swelling rate for 5% and 10% GelMA concentrations (*n* = 3 per group). **d** Successful 405 nm light-induced crosslinking of SU056 mixed with 10% GelMA. **e** Representative cross-sectional scanning electron microscopic image of 10% GelMA (scale bar as shown in image). **f** In vitro release curve of SU056 from SU056@GelMA in PBS (*n* = 5 per group). **g** In vivo live imaging showing retention of SU056 and SU056@GelMA in rat intervertebral discs at different time points. **h** Histological staining (HE, SO & FG, and Alcian Blue) of rat caudal specimens, with magnified views of the NP–AF junction and cartilage endplate. **i**, **j** Representative X-ray images of rat caudal specimens and DHI quantification (*n *= 5 biological replicates). **k–m** Representative IF images and quantification of Col2a1 and Mmp3 expression in rat NPCs (*n* = 5 biological replicates). **n** Representative HE, SO & FG, and Alcian Blue staining images of rabbit lumbar intervertebral discs from each group. **o** Representative anteroposterior and lateral X-ray images of operated rabbit lumbar segments in the SHAM, Puncture + PBS, GelMA, and SU056@GelMA groups (*n *= 5 per group). **p** Quantification of relative DHI of the operated rabbit discs. **q–s** Representative IF images of COL2A1 and MMP3 in rabbit lumbar intervertebral discs, with quantification of relative fluorescence intensity (*n *= 5 per group). Scale bars are indicated in the images. Data are presented as mean ± s.e.m. Statistical significance was determined using the indicated pairwise comparisons; NS, not significant; *P < 0.05, **P < 0.01, ***P < 0.001 (panels j, l, m, p, r and s).

Systematic optimization of the mechanical and physicochemical properties of GelMA involved comparative analyses of formulations with varying concentrations. Increasing the GelMA concentration from 5% to 10% markedly enhanced the tensile fracture strength, which is a critical parameter for potential biomaterial applications (Fig. 7a). Long-term stability assessments in PBS revealed marked differences in degradation kinetics; 5% GelMA rapidly degraded to 10.85%, whereas 10% GelMA preserved 34.65% of its initial structural integrity after 28 days (Fig. 7b). This pronounced stability profile suggests superior in vivo performance of the 10% formulation. Swelling rate analysis further substantiated the advantageous characteristics of 10% GelMA, demonstrating markedly reduced volumetric expansion compared with the 5% concentration (Fig. 7c). Consequently, we selected the 10% GelMA formulation, which offered optimal mechanical load distribution, enhanced in vivo stability, and minimized the risk of disc-associated structural compromise.

A 405 nm light-induced crosslinking strategy facilitated successful SU056@GelMA hydrogel formation (Fig. 7d), with scanning electron microscopy revealing a uniformly structured interconnected porous architecture (Fig. 7e). The in vitro drug-release assay demonstrated that SU056@GelMA facilitated a controlled and sustained release of the drug, reaching a cumulative release of 71.71% by day 35 (Fig. 7f). Drug delivery investigations compared standard SU056 injections with SU056@GelMA in rat intervertebral disc models. Live imaging provided nuanced insight into drug metabolism and distribution. Despite the limited vascularization of the disc, SU056 demonstrated a relatively rapid clearance, and complete metabolization occurred within 21 days. Conventional drug injection resulted in scattered fluorescent signals with considerable inter-tissue leakage, which is attributable to potential AF disruption. Conversely, SU056@GelMA exhibited sophisticated sustained-release characteristics with detectable drug remnants persisting for 21 days post-administration. Critically, SU056@GelMA demonstrated focused fluorescence localization, minimizing signal dispersion into adjacent tissues (Fig. 7g).

We further evaluated long-term biosafety after intradiscal implantation of PBS, GelMA, or SU056@GelMA in rat caudal discs. At 8 weeks after injection, Masson’s trichrome staining did not reveal evident collagen deposition or fibrotic encapsulation at the injection site (Supplementary Fig. 8a). IF analyses showed that α-SMA, IL-1β, IL-6, CD68/CD80, and CD68/CD206 signals in NP and AF were not increased after SU056@GelMA implantation, indicating no obvious fibrosis-associated activation, proinflammatory cytokine elevation, or macrophage polarization-associated inflammatory response under the tested conditions (Supplementary Fig. 8b–g). HE staining of the heart, liver, spleen, lung, and kidney, together with complete blood count and serum biochemical parameters, revealed no apparent systemic toxicity during the observation period (Supplementary Fig. 8h–j). These findings comprehensively validate the dual capabilities of SU056@GelMA, including sophisticated drug-release kinetics, precise spatial confinement, local histocompatibility, and systemic biosafety.

### SU056@GelMA mitigates IVDD in rat caudal and rabbit lumbar models

To comprehensively evaluate the therapeutic potential of SU056@GelMA, we first used a robust rat caudal disc needle-puncture model. Six-week-old male rats underwent precise disc puncture, followed by intranuclear injection of PBS, GelMA, or SU056@GelMA and systematic followup for pathological and molecular assessments. Multifaceted histological analyses incorporating HE, SO & FG, and Alcian Blue staining revealed nuanced tissue-remodeling dynamics (Fig. 7h). Radiographic assessments via X-ray imaging quantified disc height modifications, revealing that both GelMA and SU056@GelMA interventions effectively attenuated puncture-induced disc space narrowing with comparable efficacy (Fig. 7i, j). Puncture-induced injury precipitated marked architectural disruption of the NP, pronounced cellular attrition, fibrotic transformation, and substantial structural compromise of the AF. Comparative evaluations demonstrated that GelMA intervention partially mitigated these degenerative changes, preserving glycomatrix integrity and cellular population. The SU056@GelMA group exhibited the most promising therapeutic profile. Despite the incomplete structural reconstitution, NPCs approached a near-physiological morphology, maintaining a substantial viable cellular contingent (Fig. 7h).

IF investigations provided molecular-level mechanistic insights. Puncture treatment dramatically suppressed Col2a1 expression in NPCs. GelMA injection partially restored Col2a1 expression, whereas SU056@GelMA exhibited a significantly more pronounced rescue effect. Puncture-induced Mmp3 upregulation, which was minimally affected by GelMA, was significantly suppressed by SU056@GelMA, revealing a marked reduction compared with PBS controls (Fig. 7k–m). The expression of Cxcl8 followed a trend similar to that of Mmp3, and its puncture-induced upregulation was significantly attenuated by SU056@GelMA (Supplementary Fig. 6b). Moreover, SU056@GelMA notably alleviated degeneration-associated CD68/CD80-positive M1-like macrophage infiltration and MPO/ELA2-positive neutrophil infiltration in the rat NP (Supplementary Fig. 7b).

To strengthen the translational validation, we further examined SU056@GelMA in an NZW rabbit lumbar needle-puncture IVDD model. Compared with PBS treatment, SU056@GelMA better preserved the lumbar disc height and improved the NP and AF architecture, with reduced proteoglycan loss and less severe degenerative remodeling (Fig. 7n–p). Molecular analyses further showed that SU056@GelMA maintained COL2A1 expression and suppressed MMP3 expression in rabbit lumbar discs (Fig. 7q–s). Consistent with the YBX1–CXCL chemokine mechanism, SU056@GelMA also reduced CXCL8 expression and attenuated local CD68/CD80-positive M1-like macrophage and MPO/ELA2-positive neutrophil infiltration in the rabbit model (Supplementary Figs. 6c and 7c).

These comprehensive findings substantiate the therapeutic potential of SU056@GelMA, demonstrating its ability to intervene at multiple molecular, cellular, and tissue levels to attenuate IVDD progression in both rat caudal and rabbit lumbar IVDD models. These results highlight a promising strategy for targeted molecular intervention in degenerative disc pathology.

## Discussion

This investigation identified HDAC1, a deacetylase whose expression inversely correlates with degeneration severity, through comprehensive analysis of RNA-seq data from the Gene Expression Omnibus database and human NP specimens exhibiting various degrees of degeneration. Our gene manipulation studies, including both knockout and overexpression approaches, demonstrated that HDAC1 markedly enhances the expression of ECM synthesis markers (COL2A1 and SOX9) while concurrently suppressing ECM degradation markers (ADAMTS5 and MMP3) in NPCs, thereby serving as a critical regulator of metabolic homeostasis. Proteomic analyses revealed that HDAC1 forms a specific complex with YBX1 and modulates its protein stability. Subsequent mechanistic studies elucidated that HDAC1 facilitates YBX1 ubiquitination through deacetylation, thus accelerating its degradation via the proteasomal pathway. Functional assays confirmed that YBX1 exerts negative regulatory effects on NPC ECM metabolism by inhibiting synthesis markers while promoting degradation markers. Furthermore, RNA-seq analysis uncovered that YBX1 activates inflammatory chemokine-associated pathways, resulting in markedly upregulation of neutrophil chemokines (including *CXCL1–3*, *CXCL5*, *CXCL6*, and *CXCL8*). These molecular mechanisms were subsequently validated in HDAC1 knockout mouse models. Based on these insights, we engineered SU056@GelMA for targeted delivery of the specific YBX1 inhibitor SU056 to the intervertebral disc^47^. In rat caudal and NZW rabbit lumbar IVDD models, SU056@GelMA effectively enhanced ECM synthesis marker expression while simultaneously reducing the levels of ECM degradation markers and inflammatory chemokines, thereby attenuating IVDD progression across two complementary in vivo systems.

In oncology research, YBX1 is an established critical regulator of tumor inflammatory microenvironment formation. In lung cancer cells, YBX1 acts as a transcription factor that enhances C-C motif chemokine ligand 5 expression, thereby promoting CD8 + T cell recruitment^48,49^. Similarly, in pancreatic cancer cells, O-GlcNAc modification stimulates YBX1 phosphorylation, triggering its nuclear translocation and direct binding to the *IL-18* promoter region. This interaction augments *IL-18* transcriptional activity, subsequently mediating macrophage polarization and modulating the tumor immune microenvironment^50^. These findings collectively demonstrate the pivotal regulatory function of YBX1 in mediating interactions between tissue and immune cells. Historically, the intervertebral disc has been regarded as an immune-privileged organ owing to the protective barrier formed by the cartilaginous endplates and AF^43^. However, recent advances in single-cell sequencing technology have revealed not only heterogeneous NPC subpopulations with distinct metabolic profiles but also markedly infiltration of diverse immune cells. These observations suggest that the development and progression of inflammatory microenvironments may play a fundamental role in IVDD^44^. In our investigation, RNA-seq analysis revealed that YBX1 markedly upregulates numerous inflammatory chemokines, particularly CXCL family members with neutrophil chemotactic properties (including *CXCL1–3*, *CXCL5*, *CXCL6*, and *CXCL8*)^42^. This regulatory mechanism was subsequently confirmed through in vivo experimentation. Integrating these findings with current literature, we propose that YBX1 contributes to neutrophil infiltration and local inflammatory microenvironment formation in the intervertebral disc by regulating CXCL expression. This function not only parallels the established role of YBX1 in modulating inflammatory microenvironments in oncology but also reinforces the importance of neutrophil and inflammatory cell infiltration in IVDD pathogenesis.

During our experimental investigations, we observed that HDAC1 expression levels exert a profound influence on the subcellular localization of YBX1 in NPCs. Although YBX1 predominantly localizes to the cytoplasm under normal conditions, HDAC1 knockdown induces marked nuclear accumulation of YBX1. We propose two potential mechanisms underlying this phenomenon. First, the spatial distribution pattern of these proteins may contribute to this observation. HDAC1 primarily resides within the nucleus, whereas YBX1 exhibits a more ubiquitous distribution across both cytoplasmic and nuclear compartments. Under physiological conditions, nuclear YBX1 undergoes HDAC1-mediated degradation, whereas cytoplasmic YBX1 remains relatively stable. This differential regulation manifests as stronger YBX1 IF signals in the cytoplasm compared with the nucleus. After HDAC1 silencing, the degradation of nuclear YBX1 is inhibited, resulting in nuclear accumulation and consequently enhanced fluorescence intensity within this compartment. Second, HDAC1 may directly regulate YBX1 nuclear translocation. Previous studies have established that YBX1 predominantly localizes to the cytoplasm in multiple cell types but can translocate to the nucleus in response to specific stimuli. For instance, in pancreatic cancer cells, O-GlcNAcylation promotes YBX1 phosphorylation, inducing its nuclear translocation where it functions as a transcription factor binding to the *IL-18* promoter region to enhance transcription^50^. Similarly, in human embryonic lung fibroblasts, transforming growth factor-β stimulation triggers YBX1 nuclear translocation, enabling its subsequent binding to the *COL1A1* promoter to upregulate *COL1A1* expression^51^. Protein phosphorylation represents a well-established regulatory mechanism governing nuclear translocation. Beyond the aforementioned phosphorylation-dependent nuclear translocation of YBX1 in pancreatic cancer cells, signal transducer and activator of transcription 3 phosphorylation in NPCs has been demonstrated to facilitate its nuclear entry and subsequent transcriptional activity^52^. Integrating existing literature with our experimental observations, we hypothesize that specific phosphorylation sites on YBX1 may regulate its subcellular localization, and that this phosphorylation status may be modulated by HDAC1-mediated deacetylation. Future investigations will explore whether PTMs directly influence the spatial distribution and translocation dynamics of YBX1.

This study makes several important contributions to the field by elucidating the pivotal role of the HDAC1–YBX1 axis in IVDD. Our findings substantially expand the current understanding of PTM regulatory mechanisms operating within NPCs and provide a promising biomaterial-based therapeutic approach for clinical intervention. The identification of YBX1 as a central regulatory node in both ECM metabolism and inflammatory microenvironment formation offers new avenues for developing targeted treatments for degenerative disc disease.

**Fig. 8:**
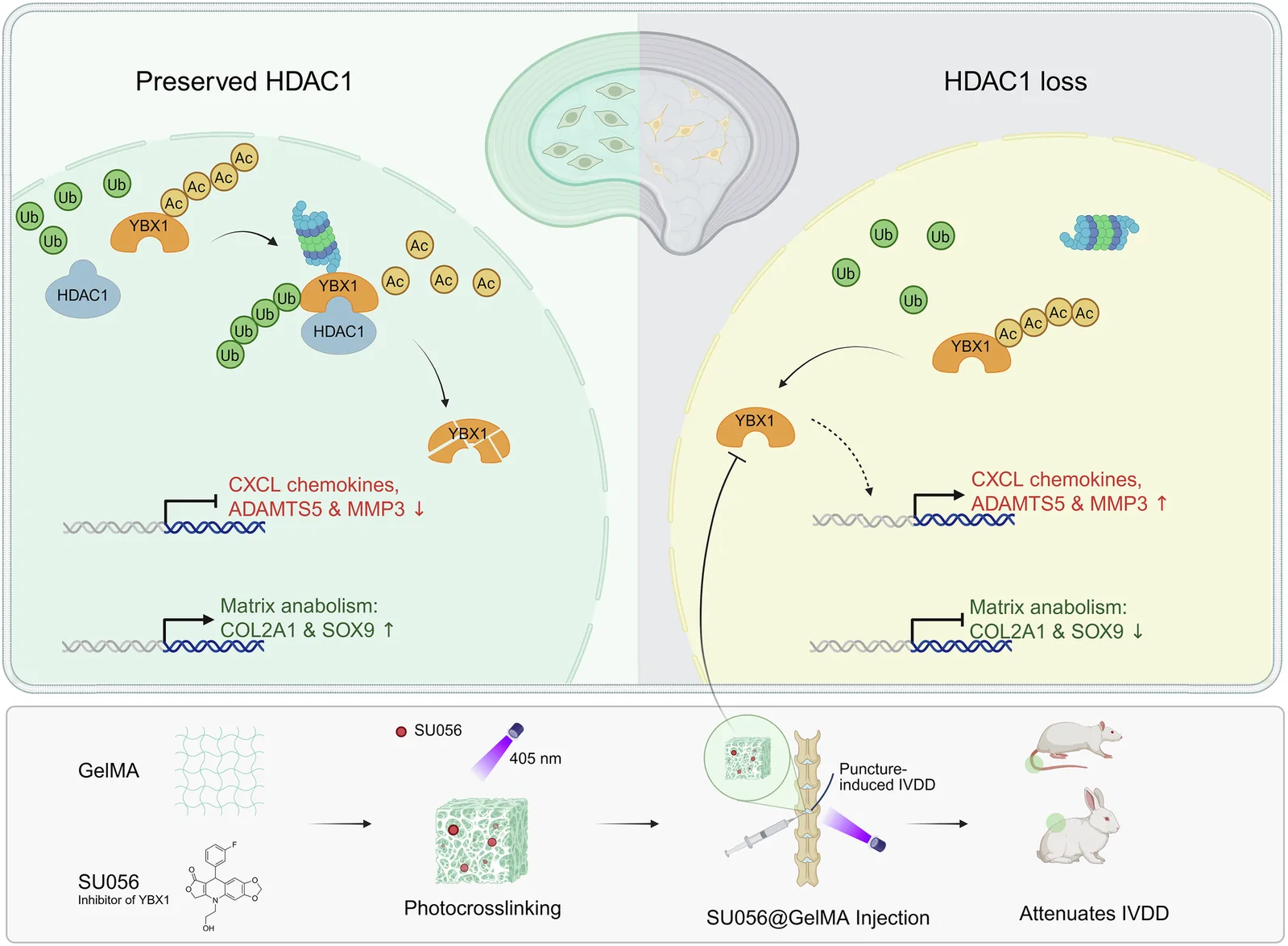
Mechanism of HDAC1 and YBX1 in NP cells, as well as the synthesis of SU056@GelMA and its therapeutic effect in an intervertebral disc degeneration model. Created in BioRender. Ma, J. (2026) https://BioRender.com/0puv224.

In conclusion, decreasing HDAC1 levels disrupt NPC homeostasis by reducing YBX1 deacetylation and degradation during IVDD. Accumulated YBX1 impairs ECM balance and activates chemokine networks, particularly *CXCLs*, which recruit neutrophils. This dual mechanism simultaneously compromises ECM integrity and promotes inflammation (Fig. 8). Furthermore, targeting the HDAC1–YBX1 axis using SU056@GelMA effectively rescued IVDD phenotypes in both rat caudal and NZW rabbit lumbar models.

## Supplementary information

Supplementary information

## Data Availability

All data needed to evaluate the conclusions in the paper are available in the paper, the supplementary materials, and the NCBI Gene Expression Omnibus (GEO). The RNA-sequencing data generated in this study have been deposited in GEO under accession number GSE344516. Additional data related to this paper may be requested from the authors.

## References

[1] Driscoll, T. et al. The global burden of occupationally related low back pain: estimates from the Global Burden of Disease 2010 study. *Ann. Rheum. Dis.* **73**, 975–981 (2014). 10.1136/annrheumdis-2013-204631 24665117

[2] Katz, J. N. Lumbar disc disorders and low-back pain: socioeconomic factors and consequences. *J. Bone. Joint. Surg. Am.* **88**, 21–24 (2006). 10.2106/JBJS.E.01273 16595438

[3] Liang, H. et al. The proteolysis of ECM in intervertebral disc degeneration. Int. J. Mol. Sci. **23**, 1715 (2022). 10.3390/ijms23031715 PMC8835917 35163637

[4] Lyu, F. J. et al. Painful intervertebral disc degeneration and inflammation: from laboratory evidence to clinical interventions. *Bone. Res.* **9**, 7 (2021). 10.1038/s41413-020-00125-x PMC7846842 33514693

[5] Zhu, D. et al. Altered metabolism and inflammation driven by post-translational modifications in intervertebral disc degeneration. *Research (Wash D C).* **7**, 0350 (2024). 10.34133/research.0350 PMC10997488 38585329

[6] Qingxin, S. et al. Programmable DNA hydrogel provides suitable microenvironment for enhancing autophagy-based therapies in intervertebral disc degeneration treatment. *J. Nanobiotechnology.* **21**, 350 (2023). 10.1186/s12951-023-02109-5 PMC10537074 37759249

[7] Gonzalez, A., Hall, M. N., Lin, S. C. & Hardie, D. G. AMPK and TOR: the yin and yang of cellular nutrient sensing and growth control. *Cell. Metab.* **31**, 472–492 (2020). 10.1016/j.cmet.2020.01.015 32130880

[8] Zhang, W. et al. Disassembly of the TRIM56-ATR complex promotes cytoDNA/cGAS/STING axis-dependent intervertebral disc inflammatory degeneration. J. Clin. Invest. **134**, e165140 (2024). 10.1172/JCI165140 PMC10940101 38488012

[9] Drazic, A., Myklebust, L. M., Ree, R. & Arnesen, T. The world of protein acetylation. *Biochim. Biophys. Acta.* **1864**, 1372–1401 (2016). 10.1016/j.bbapap.2016.06.007 27296530

[10] Shvedunova, M. & Akhtar, A. Modulation of cellular processes by histone and non-histone protein acetylation. *Nat. Rev. Mol. Cell. Biol.* **23**, 329–349 (2022). 10.1038/s41580-021-00441-y 35042977

[11] Narita, T., Weinert, B. T. & Choudhary, C. Functions and mechanisms of non-histone protein acetylation. *Nat. Rev. Mol. Cell. Biol.* **20**, 156–174 (2019). 10.1038/s41580-018-0081-3 30467427

[12] Fu, B. et al. Matrix stiffening promotes chondrocyte senescence and the osteoarthritis development through downregulating HDAC3. *Bone. Res.* **12**, 32 (2024). 10.1038/s41413-024-00333-9 PMC11126418 38789434

[13] Ji, M. L. et al. Sirt6 attenuates chondrocyte senescence and osteoarthritis progression. *Nat. Commun.* **13**, 7658 (2022). 10.1038/s41467-022-35424-w PMC9741608 36496445

[14] Yang, X. J. & Seto, E. Lysine acetylation: codified crosstalk with other posttranslational modifications. *Mol. Cell.* **31**, 449–461 (2008). 10.1016/j.molcel.2008.07.002 PMC2551738 18722172

[15] Lee, J. M., Hammaren, H. M., Savitski, M. M. & Baek, S. H. Control of protein stability by post-translational modifications. *Nat. Commun.* **14**, 201 (2023). 10.1038/s41467-023-35795-8 PMC9839724 36639369

[16] Geffen, Y. et al. Pan-cancer analysis of post-translational modifications reveals shared patterns of protein regulation. Cell **186**, 3945–3967 (2023). 10.1016/j.cell.2023.07.013 PMC10680287 37582358

[17] Shimizu, K. et al. Interplay between protein acetylation and ubiquitination controls MCL1 protein stability. *Cell. Rep.* **37**, 109988 (2021). 10.1016/j.celrep.2021.109988 PMC8621139 34758305

[18] Gong, Z. et al. CircRREB1 mediates lipid metabolism related senescent phenotypes in chondrocytes through FASN post-translational modifications. *Nat. Commun.* **14**, 5242 (2023). 10.1038/s41467-023-40975-7 PMC10462713 37640697

[19] Jia, S. et al. Single-cell sequencing reveals cellular heterogeneity of nucleus pulposus in intervertebral disc degeneration. *Sci. Rep.* **14**, 27245 (2024). 10.1038/s41598-024-78675-x PMC11549379 39516278

[20] Hu, X. et al. Single-cell sequencing: New insights for intervertebral disc degeneration. *Biomed. Pharmacother.* **165**, 115224 (2023). 10.1016/j.biopha.2023.115224 37516017

[21] Urrutia, J. et al. The Pfirrmann classification of lumbar intervertebral disc degeneration: an independent inter- and intra-observer agreement assessment. *Eur. Spine. J.* **25**, 2728–2733 (2016). 10.1007/s00586-016-4438-z 26879918

[22] Huang, Y. et al. circSPG21 protects against intervertebral disc disease by targeting miR-1197/ATP1B3. *Exp. Mol. Med.* **53**, 1547–1558 (2021). 10.1038/s12276-021-00674-z PMC8568895 34611269

[23] Wang, J. et al. Small molecule natural compound agonist of SIRT3 as a therapeutic target for the treatment of intervertebral disc degeneration. *Exp. Mol. Med.* **50**, 1–14 (2018). 10.1038/s12276-018-0173-3 PMC6232087 30420619

[24] Huang, Y. et al. Inhibition of intervertebral disc disease progression via the circPKNOX1-miR-370-3p-KIAA0355 axis. *Cell. Death. Discov.* **7**, 39 (2021). 10.1038/s41420-021-00420-4 PMC7910476 33637685

[25] Tang, D. et al. SRplot: a free online platform for data visualization and graphing. *PloS One.* **18**, e0294236 (2023). 10.1371/journal.pone.0294236 PMC10635526 37943830

[26] Lyu, F. et al. OmicStudio: a composable bioinformatics cloud platform with real-time feedback that can generate high-quality graphs for publication. *Imeta* **2**, e85 (2023). 10.1002/imt2.85 PMC10989813 38868333

[27] Masuda, K. et al. A novel rabbit model of mild, reproducible disc degeneration by an anulus needle puncture: correlation between the degree of disc injury and radiological and histological appearances of disc degeneration. *Spine* **30**, 5–14 (2005). 10.1097/01.brs.0000148152.04401.20 15626974

[28] Luo, T. D. et al. A percutaneous, minimally invasive annulus fibrosus needle puncture model of intervertebral disc degeneration in rabbits. *J. Orthop. Surg.* **26**, 2309499018792715 (2018). 10.1177/2309499018792715 30114959

[29] Garber, J. C. et al. *Guide for the Care and Use of Laboratory Animals: Eight Edition*. The National Academies Press, 2011. 21595115

[30] Hu, J. et al. AQP3-liposome@GelMA promotes overloaded-induced degenerated disc regeneration via IBSP/ITG alphaVbeta3/AKT pathway. *Int. J. Biol. Macromol.* **292**, 139238 (2025). 10.1016/j.ijbiomac.2024.139238 39733899

[31] Wei, Q. et al. MiR-17-5p-engineered sEVs encapsulated in GelMA hydrogel facilitated diabetic wound healing by targeting PTEN and p21. *Adv. Sci. (Weinh)* **11**, e2307761 (2024). 10.1002/advs.202307761 PMC10987139 38286650

[32] Tian, R. et al. MSdb: an integrated expression atlas of human musculoskeletal system. *iScience* **26**, 106933 (2023). 10.1016/j.isci.2023.106933 PMC10291471 37378342

[33] Li, Z., Sun, Y., He, M. & Liu, J. Differentially-expressed mRNAs, microRNAs and long noncoding RNAs in intervertebral disc degeneration identified by RNA-sequencing. *Bioengineered* **12**, 1026–1039 (2021). 10.1080/21655979.2021.1899533 PMC8806213 33764282

[34] Farhang, N. et al. Lentiviral CRISPR epigenome editing of inflammatory receptors as a gene therapy strategy for disc degeneration. *Hum. Gene. Ther.* **30**, 1161–1175 (2019). 10.1089/hum.2019.005 PMC6761595 31140325

[35] Bydon, M. et al. Lumbar intervertebral disc mRNA sequencing identifies the regulatory pathway in patients with disc herniation and spondylolisthesis. *Gene* **750**, 144634 (2020). 10.1016/j.gene.2020.144634 32240779

[36] Lorenzi, L. et al. The RNA Atlas expands the catalog of human non-coding RNAs. *Nat. Biotechnol.* **39**, 1453–1465 (2021). 10.1038/s41587-021-00936-1 34140680

[37] Wu, Y. et al. Metabolite asymmetric dimethylarginine (ADMA) functions as a destabilization enhancer of SOX9 mediated by DDAH1 in osteoarthritis. *Sci. Adv.* **9**, eade5584 (2023). 10.1126/sciadv.ade5584 PMC9908022 36753544

[38] Miao, Y. et al. Cycloheximide (CHX) chase assay to examine protein half-life. *Bio. Protoc.* **13**, e4690 (2023). 10.21769/BioProtoc.4690 PMC10262206 37323633

[39] Noitem, R., Yuajit, C., Soodvilai, S., Muanprasat, C. & Chatsudthipong, V. Steviol slows renal cyst growth by reducing AQP2 expression and promoting AQP2 degradation. *Biomed. Pharmacother.* **101**, 754–762 (2018). 10.1016/j.biopha.2018.02.139 29524884

[40] Locati, M. & Murphy, P. M. Chemokines and chemokine receptors: biology and clinical relevance in inflammation and AIDS. *Annu. Rev. Med.* **50**, 425–440 (1999). 10.1146/annurev.med.50.1.425 10073287

[41] Kobayashi, Y. Neutrophil infiltration and chemokines. *Crit. Rev. Immunol.* **26**, 307–316 (2006). 10.1615/critrevimmunol.v26.i4.20 17073556

[42] Kolaczkowska, E. & Kubes, P. Neutrophil recruitment and function in health and inflammation. *Nat. Rev. Immunol.* **13**, 159–175 (2013). 10.1038/nri3399 23435331

[43] Sun, Z., Liu, B. & Luo, Z. J. The immune privilege of the intervertebral disc: implications for intervertebral disc degeneration treatment. *Int. J. Med. Sci.* **17**, 685–692 (2020). 10.7150/ijms.42238 PMC7085207 32210719

[44] Li, W. et al. Deciphering the sequential changes of monocytes/macrophages in the progression of IDD with longitudinal approach using single-cell transcriptome. *Front. Immunol.* **14**, 1090637 (2023). 10.3389/fimmu.2023.1090637 PMC9929188 36817437

[45] Wang, L. et al. Revealing the immune infiltration landscape and identifying diagnostic biomarkers for lumbar disc herniation. *Front. Immunol.* **12**, 666355 (2021). 10.3389/fimmu.2021.666355 PMC8190407 34122424

[46] Zhang, T. L. et al. Single-cell RNA sequencing reveals the MIF/ACKR3 receptor-ligand interaction between neutrophils and nucleus pulposus cells in intervertebral disc degeneration. *Transl. Res.* **272**, 1–18 (2024). 10.1016/j.trsl.2024.05.011 38823438

[47] Desai, S. U., Srinivasan, S. S., Kumbar, S. G. & Moss, I. L. Hydrogel-based strategies for intervertebral disc regeneration: advances, challenges and clinical prospects. *Gels* **10**, 62 (2024). 10.3390/gels10010062 PMC10815657 38247785

[48] Li, R. et al. The recruitment of CD8(+) T cells through YBX1 stabilization abrogates tumor intrinsic oncogenic role of MIR155HG in lung adenocarcinoma. *Cell. Death. Discov.* **10**, 334 (2024). 10.1038/s41420-024-02102-3 PMC11266398 39043648

[49] Song, S. et al. A novel long noncoding RNA, TMEM92-AS1, promotes gastric cancer progression by binding to YBX1 to mediate CCL5. *Mol. Oncol.* **15**, 1256–1273 (2021). 10.1002/1878-0261.12863 PMC8024739 33247987

[50] Zhang, H. R. et al. The GFPT2-O-GlcNAcylation-YBX1 axis promotes IL-18 secretion to regulate the tumor immune microenvironment in pancreatic cancer. *Cell. Death. Dis.* **15**, 244 (2024). 10.1038/s41419-024-06589-7 PMC10995196 38575607

[51] Sun, W., Hou, F., Panchenko, M. P. & Smith, B. D. A member of the Y-box protein family interacts with an upstream element in the alpha1(I) collagen gene. *Matrix. Biol.* **20**, 527–541 (2001). 10.1016/s0945-053x(01)00163-9 11731270

[52] Chen, L. et al. Endoplasmic reticulum stress exacerbates nucleus pulposus cell pyroptosis via PERK-dependent activation of JAK1-STAT3 signaling. *Cell. Biochem. Funct.* **43**, e70148 (2025). 10.1002/cbf.70148 41307399

