# Supplementary material for: HDAC1 maintains metabolic homeostasis in nucleus pulposus cells via post-translational modification interactions with YBX1
Source: Exp Mol Med. 2026 Sep 9;58(9):3097–112. doi: 10.1038/s12276-026-01843-8 (PMC13633268; doi:10.1038/s12276-026-01843-8)
Supplement: Supplementary file 1 [file 12276_2026_1843_MOESM1_ESM.pdf]

Supplementary Materials for

HDAC1 Maintains Metabolic Homeostasis in Nucleus Pulposus Cells via Post-translational Modification Interactions with YBX1

Bohao Zhou *et al.*

*Corresponding author. Email: sealteam@zju.edu.cn

Supplementary Text

a

	ShampleName	ProjectID	Disease	Tissue Condition	SourceTissue Celltype	Age	Gender
1	GSM5098917	GSE167199	Healthy	Normal	Nucleus_pulposus_tissue	29	N.A.
2	GSM5098915	GSE167199	Healthy	Normal	Nucleus_pulposus_tissue	31	N.A.
3	GSM5098913	GSE167199	Healthy	Normal	Nucleus_pulposus_tissue	35	N.A.
4	GSM4117763	GSE138734	Healthy	Normal	Nucleus_pulposus_cells	N.A.	N.A.
5	GSM4117765	GSE138734	Healthy	Normal	Nucleus_pulposus_cells	N.A.	N.A.
6	GSM4409347	GSE146904	Degenerative_spine_disease	Pathological	Nucleus_pulposus_tissue	45	Male
7	GSM4409351	GSE146904	Degenerative_spine_disease	Pathological	Nucleus_pulposus_tissue	49	Male
8	GSM5098919	GSE167199	Degenerative_spine_disease	Pathological	Nucleus_pulposus_tissue	57	N.A.
9	GSM3897507	GSE132958	Degenerative_spine_disease	Pathological	Nucleus_pulposus_cells	26	Male
10	GSM3897508	GSE132958	Degenerative_spine_disease	Pathological	Nucleus_pulposus_cells	26	Male

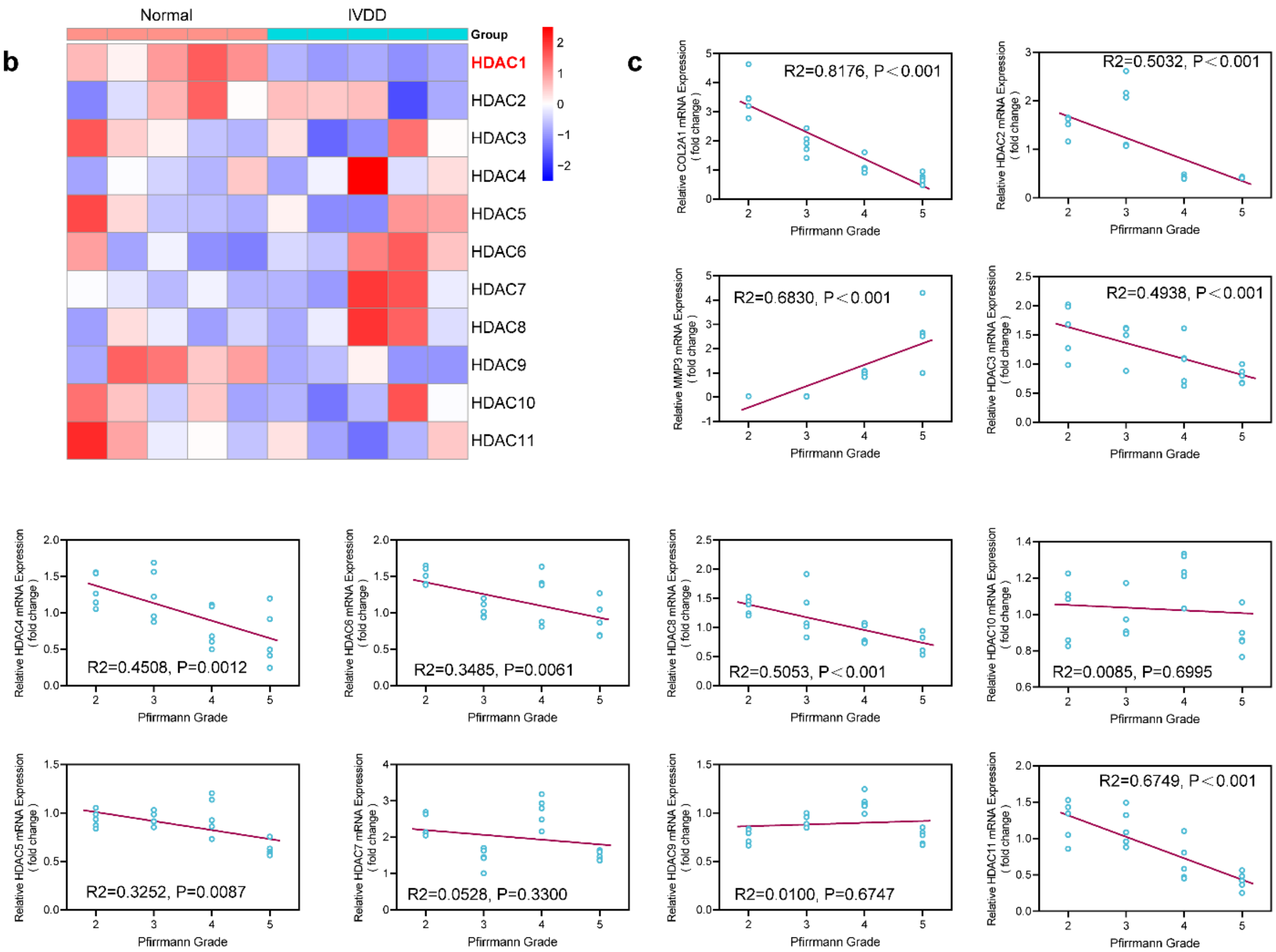

Supplementary Fig. 1. (a) RNA-seq sample classification: tissues were grouped into Normal and Pathological (n=5 per group). (b) Heatmap of HDAC expression in normal vs. degenerated NP tissues based on RNA-seq (n=5 per group). (c) Transcriptional changes in COL2A1, MMP3, and HDAC2-11 in NPCs across Pfirrmann Grades II-V (n=5 per group).

Correlation analysis was performed using simple linear regression (a).

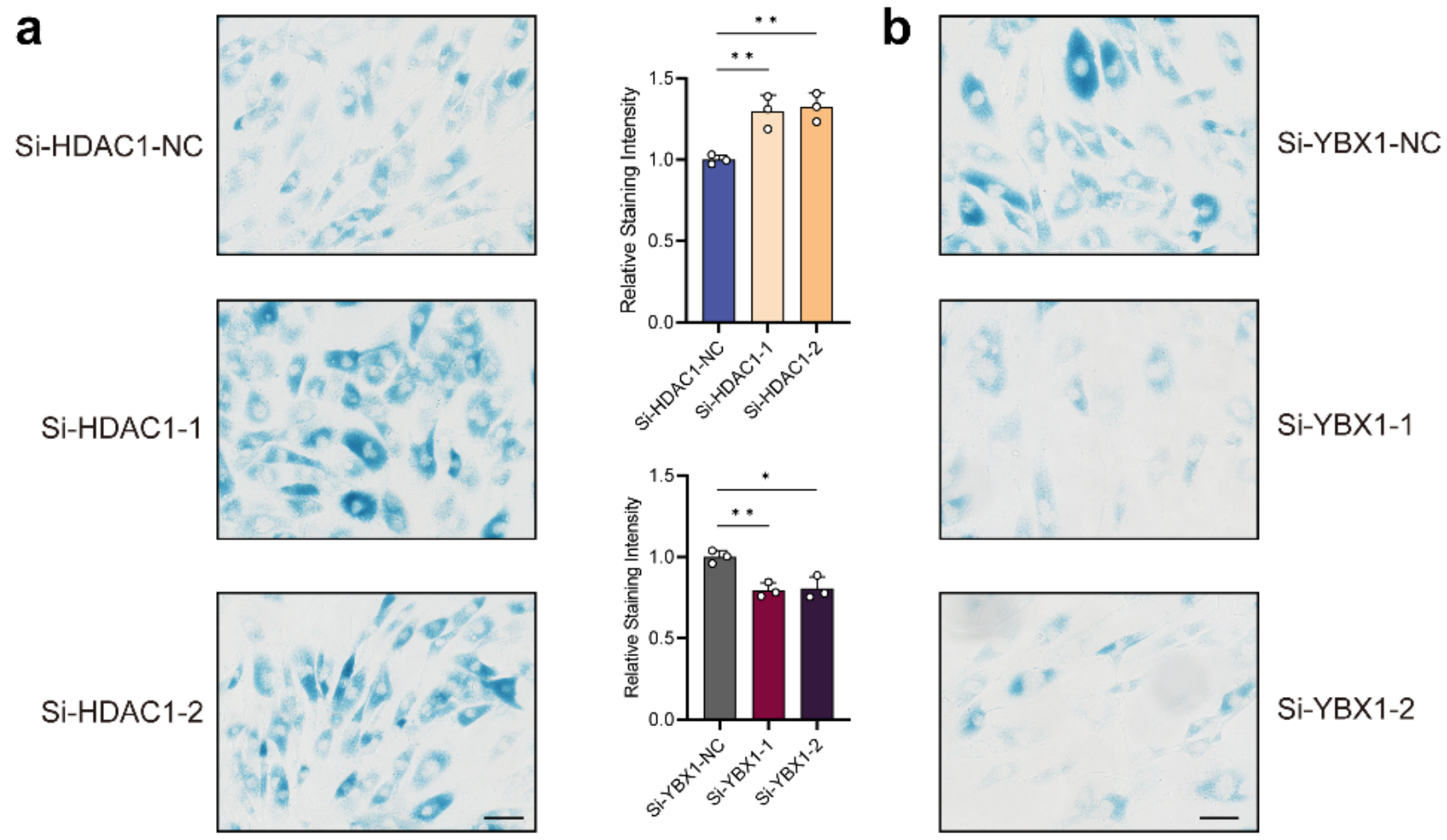

Supplementary Fig. 2. (a) β-gal staining showing increased staining intensity in HDAC1-silenced NPCs (n = 3 biological replicates). Scale bar: 50 µm. (b) β-gal staining showing decreased staining intensity in YBX1-silenced NPCs (n = 3 biological replicates). Scale bar: 50 µm.

Data are presented as mean ± SEM. Statistical significance was determined by an unpaired t-test: *P < 0.05, **P < 0.01, ***P < 0.001 (a and b).

a

Sequence	Mass	Leading razor protein	Score
NEGSESAPEGQAQQR	1586.69	sp|P62961|YBOX1_RAT	354.69
EDGNEEDKENQGDETQGQQPPQR	2627.10	sp|P62961|YBOX1_RAT	318.66
NYQQNYQNSESGEK	1687.71	sp|P62961|YBOX1_RAT	281.41
EDVFVHQTAIK	1285.67	sp|P62961|YBOX1_RAT	265.67
GAEAANVTGPGGVPVQGSK	1694.86	sp|P62961|YBOX1_RAT	225.55
NYQQNYQNSESGEKNEGSESAPEGQAQQR	3256.39	sp|P62961|YBOX1_RAT	199.1
NDTKEDVFVHQTAIK	1743.88	sp|P62961|YBOX1_RAT	198.23
RPQYSNPPVQGEVMEGADNQGAGEQGRPVR	3222.52	sp|P62961|YBOX1_RAT	162.91
SVGDGETVEFDVVEGEK	1794.82	sp|P62961|YBOX1_RAT	160.69
PGSTGSGAGSGGPGGLTSAAPAGGDKK	2198.06	sp|P62961|YBOX1_RAT	142.14
AADPPAENSSAPEAEQGGAE	1896.80	sp|P62961|YBOX1_RAT	136.72
RPQYSNPPVQGEVMEGADNQGAGEQGR	2870.30	sp|P62961|YBOX1_RAT	134.81
SSEAETQQPPAAPAAALSAADTKPGSTGSGAGSGGPGGLTSAAPAGGDK	4263.02	sp|P62961|YBOX1_RAT	99.794
SSEAETQQPPAAPAAALSAADTKPGSTGSGAGSGGPGGLTSAAPAGGDKK	4391.11	sp|P62961|YBOX1_RAT	89.509
RPENPKPQDGK	1264.65	sp|P62961|YBOX1_RAT	88.021

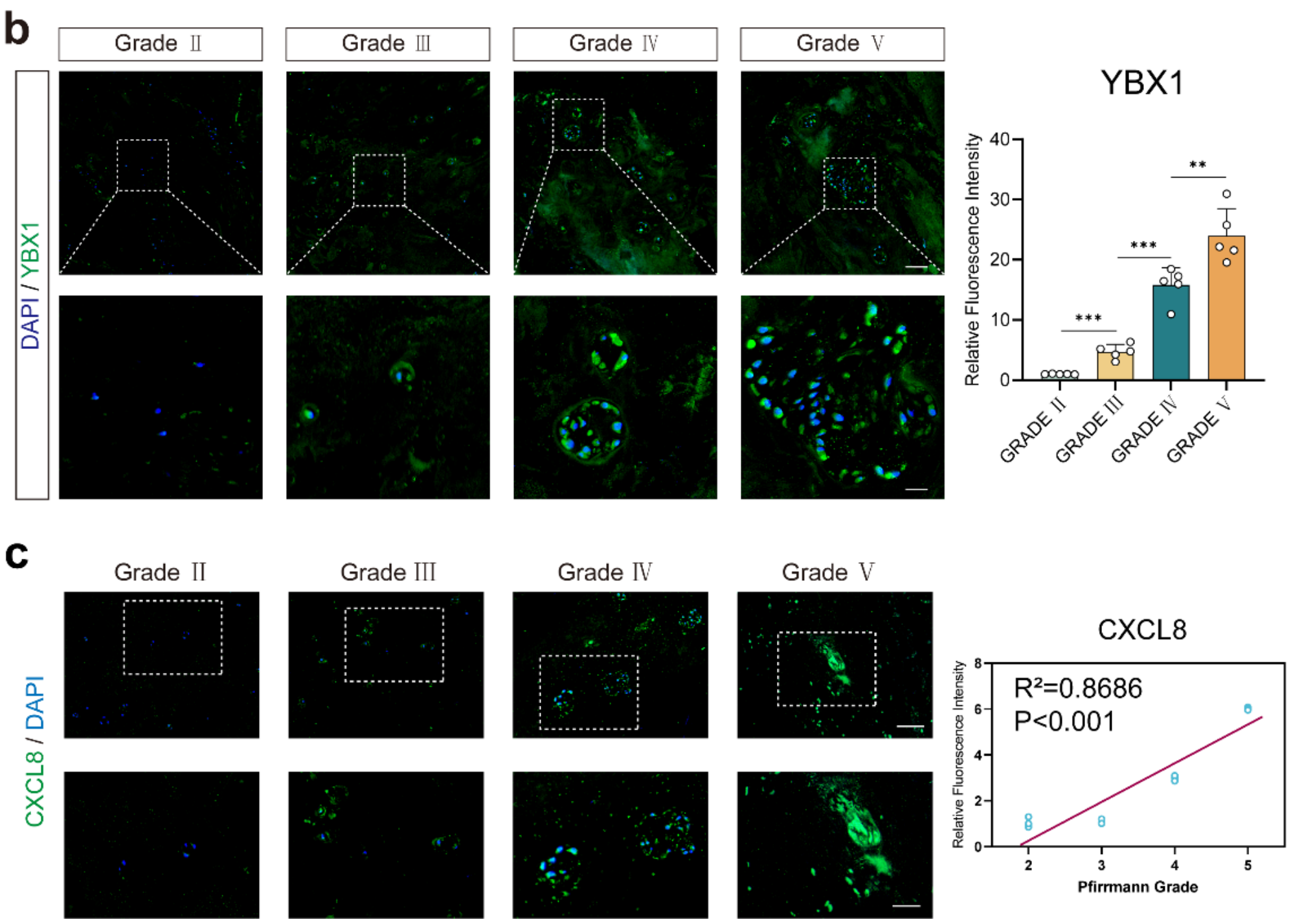

Supplementary Fig. 3. (a) MS results showing enriched YBX1-derived peptides. (b) IF staining of YBX1 in NP tissues, with fluorescence intensity quantification (grades II–V, n = 5 per group). Scale bars: low magnification 100 μm; high magnification 25 μm. (c) IF staining of CXCL8 in NP tissues, with fluorescence intensity quantification (grades II–V, n = 5 per group). Scale bars: low magnification 100 μm; high magnification 50 μm.

Data presented as mean ± SEM. Statistical differences calculated using unpaired t-test, where *P < 0.05, **P < 0.01, ***P < 0.001 (panels b). Correlation analyses performed using simple linear regression (panels c).

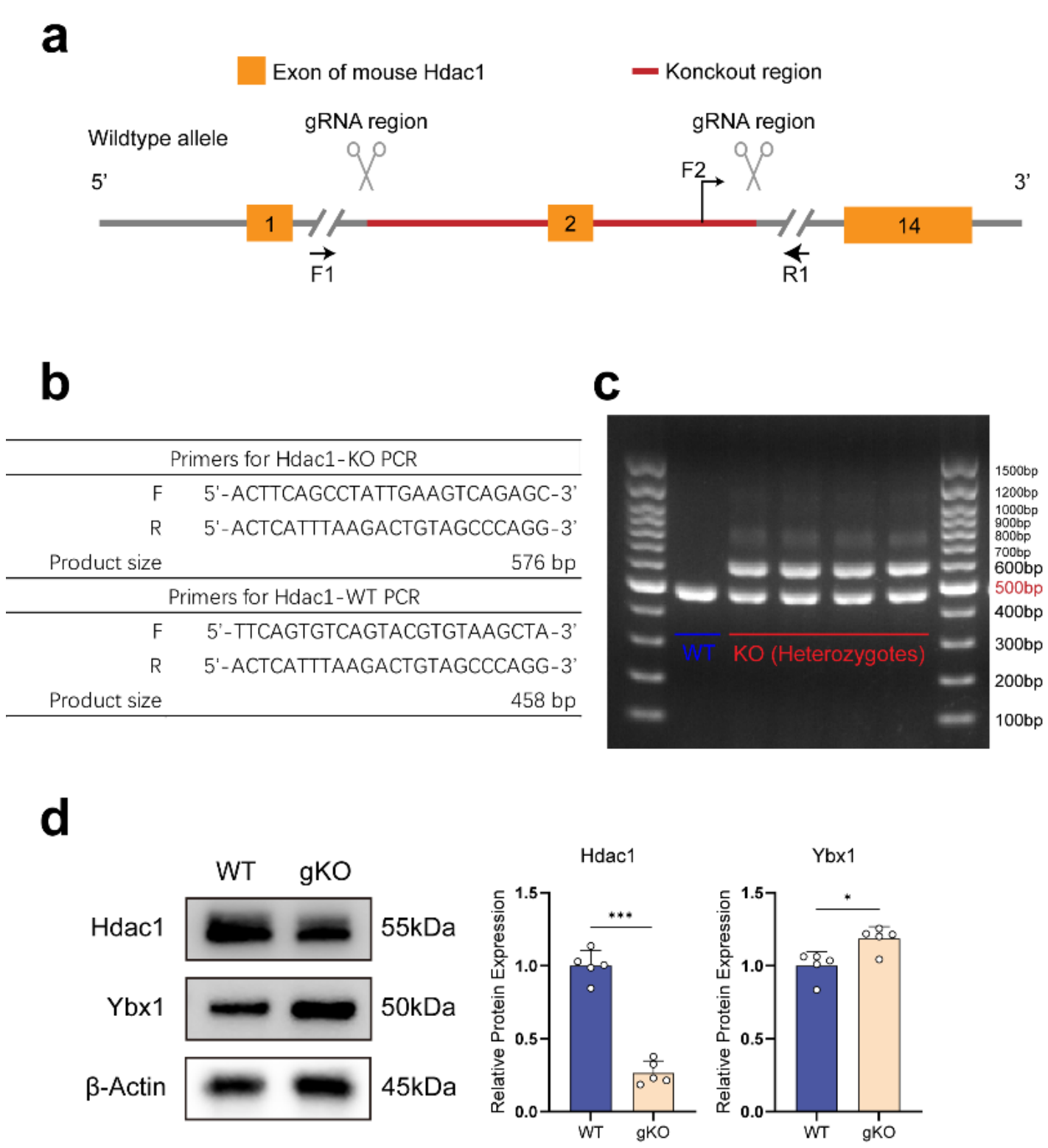

Supplementary Fig. 4. (a) Schematic representation of the HDAC1 gKO gene structure. (b) Primer design and expected product sizes for genetic identification of knockout mice. (c) Representative genetic identification results of selected mice. WT mice display a single 458 bp band, while KO mice exhibit both a 576 bp and a 458 bp band. (d) Expression of Hdac1 and Ybx1 in NPCs of mice (n = 5 biological replicates).

Data presented as mean ± SEM. Statistical differences calculated using unpaired t-test, where *P < 0.05, **P < 0.01, ***P < 0.001 (panel d).

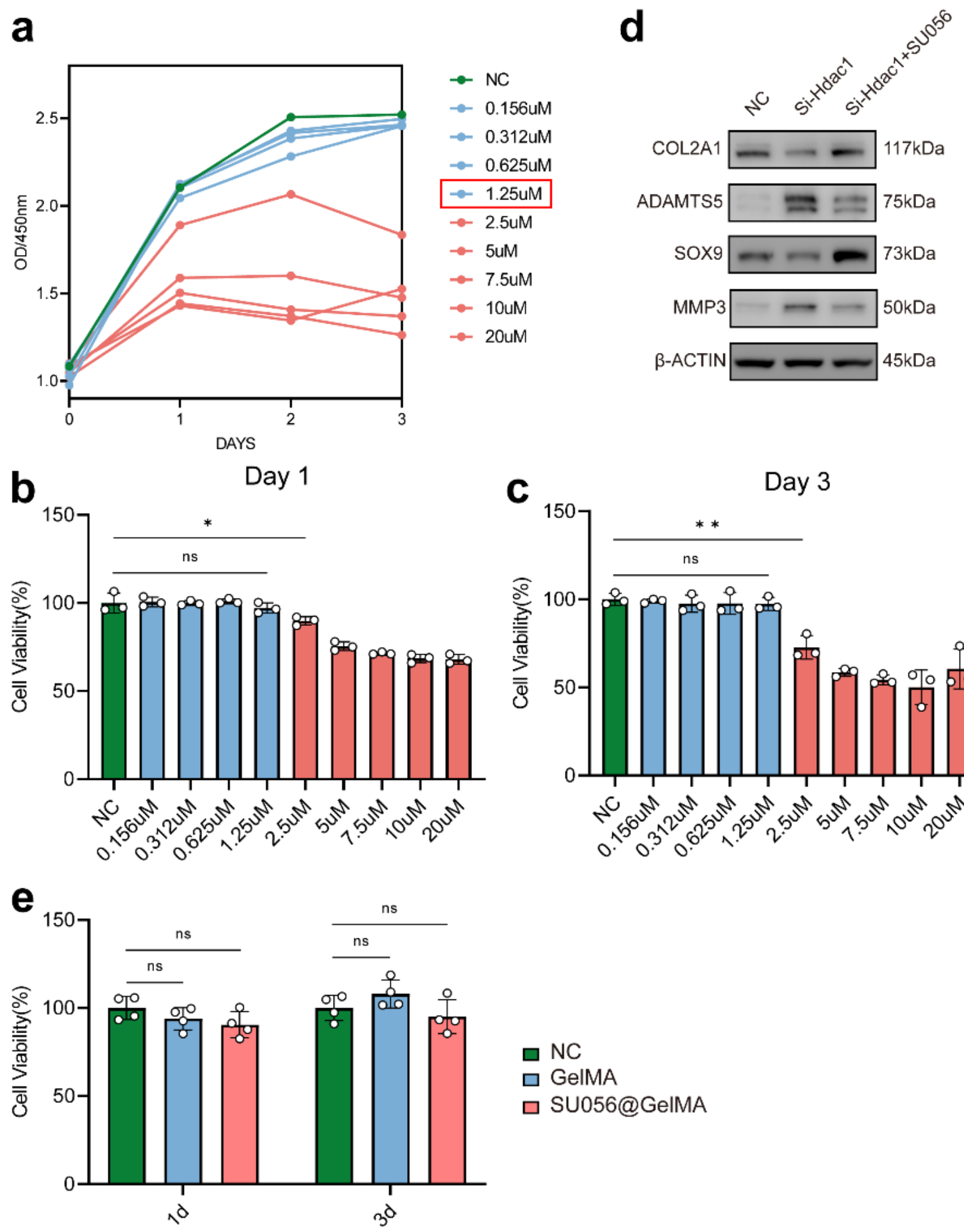

Supplementary Fig. 5. (a-c) Proliferation and viability curves of rat NPCs treated with different SU056 concentrations (n = 3 biological replicates). (d) Expression of ECM degradation markers in rat NPCs under three conditions: untreated, HDAC1 knockdown, and HDAC1 knockdown + 1.25 μM SU056 (n = 3 biological replicates). (e) Cell viability assay of rat NPCs treated with GelMA alone or SU056@GelMA, assessed on Day 1 and Day 3 (n = 4 biological replicates).

Data are presented as mean ± SEM. Statistical significance was determined by an unpaired t-test: *P < 0.05, **P < 0.01, ***P < 0.001 (panels b, c, and e).

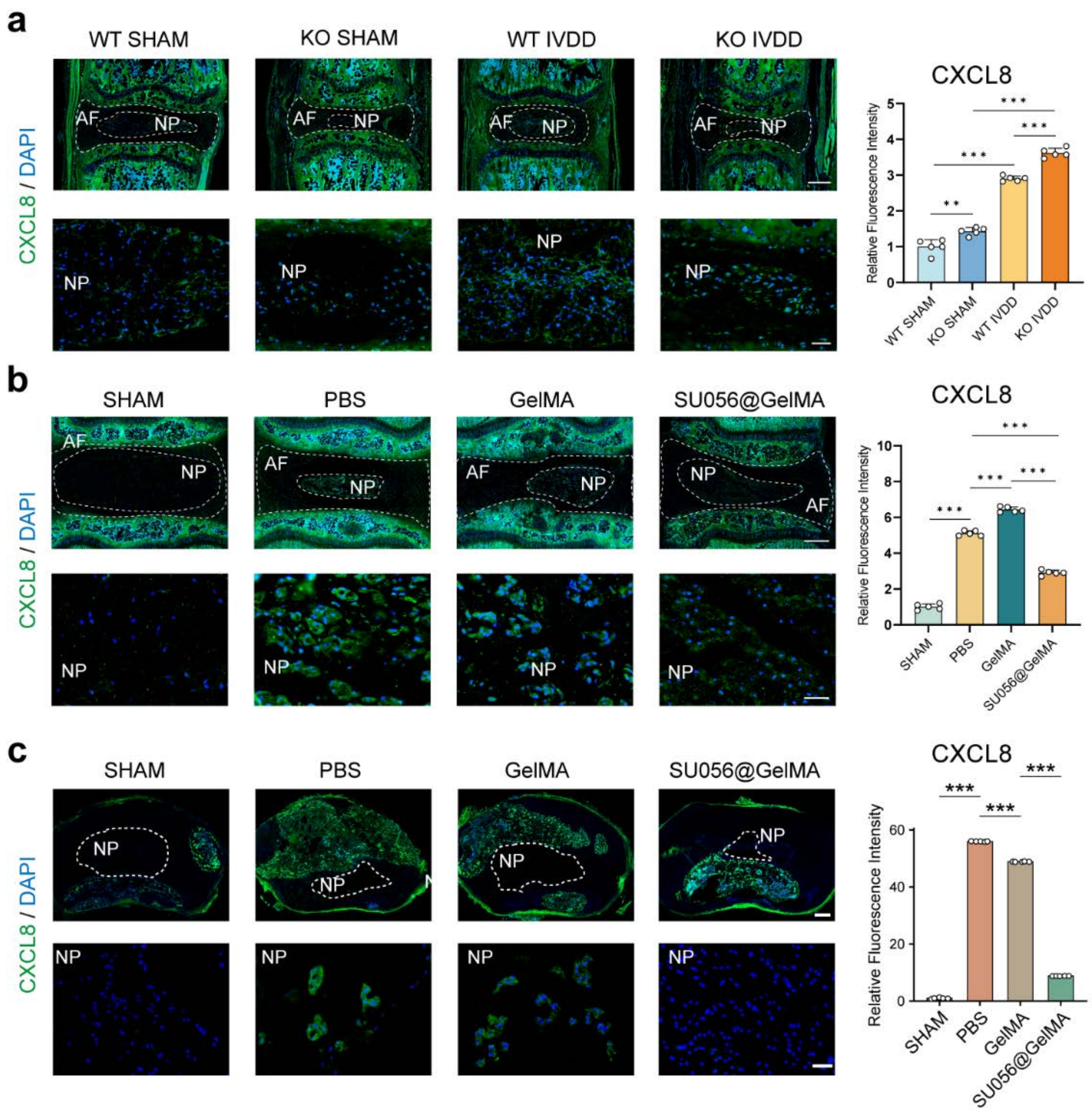

Supplementary Fig. 6. CXCL8 expression in intervertebral discs after genetic or hydrogel-based intervention. (a) IF images showing Cxcl8 expression in the nucleus pulposus after Hdac1 KO and disc puncture. Scale bars: low magnification 300 μm; high magnification 60 μm. (b) Representative IF images showing Cxcl8 expression levels in rat NPCs after puncture and hydrogel injection, with fluorescence intensity quantification (n = 5 biological replicates). Scale bars: low magnification 500 μm; high magnification 50 μm. (c) Representative IF images of CXCL8 in New Zealand White rabbit lumbar intervertebral discs from the SHAM, PBS, GelMA, and SU056@GelMA groups, with quantification of relative fluorescence intensity (n = 5 per group). Scale bars are indicated in the images.

Data are presented as mean ± SEM. Statistical significance was determined using the indicated pairwise comparisons; NS, not significant; *P < 0.05, **P < 0.01, ***P < 0.001 (panels a, b and c).

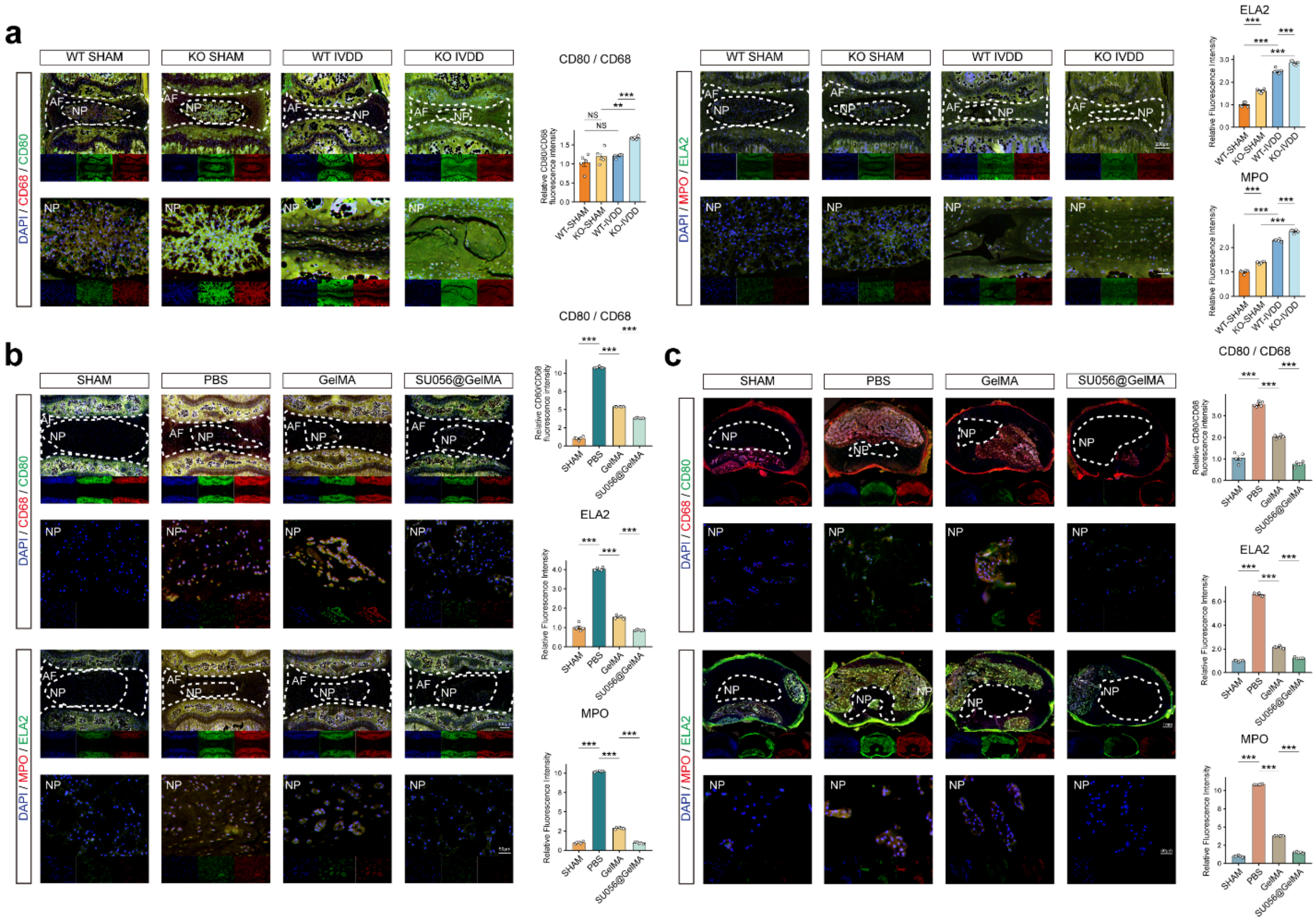

Supplementary Fig. 7. Assessment of local M1-like macrophage and neutrophil infiltration in intervertebral discs. (a) IF images demonstrating CD68/CD80-positive M1-like macrophage infiltration and MPO/ELA2-positive neutrophil infiltration in mouse nucleus pulposus tissue following puncture, with quantitative analysis (n = 5 biological replicates). (b) IF images demonstrating CD68/CD80-positive M1-like macrophage infiltration and MPO/ELA2-positive neutrophil infiltration in rat nucleus pulposus tissue following puncture and hydrogel injection, with quantitative analysis (n = 5 biological replicates). (c) Representative IF images of CD68/CD80 and MPO/ELA2 in New Zealand White rabbit lumbar intervertebral discs from the SHAM, PBS, GelMA, and SU056@GelMA groups, with quantification of relative fluorescence intensity (n = 5 per group). Scale bars are indicated in the images.

Data are presented as mean ± SEM. Statistical significance was determined using the indicated pairwise comparisons; NS, not significant; *P < 0.05, **P < 0.01, ***P < 0.001 (panels a, b and c).

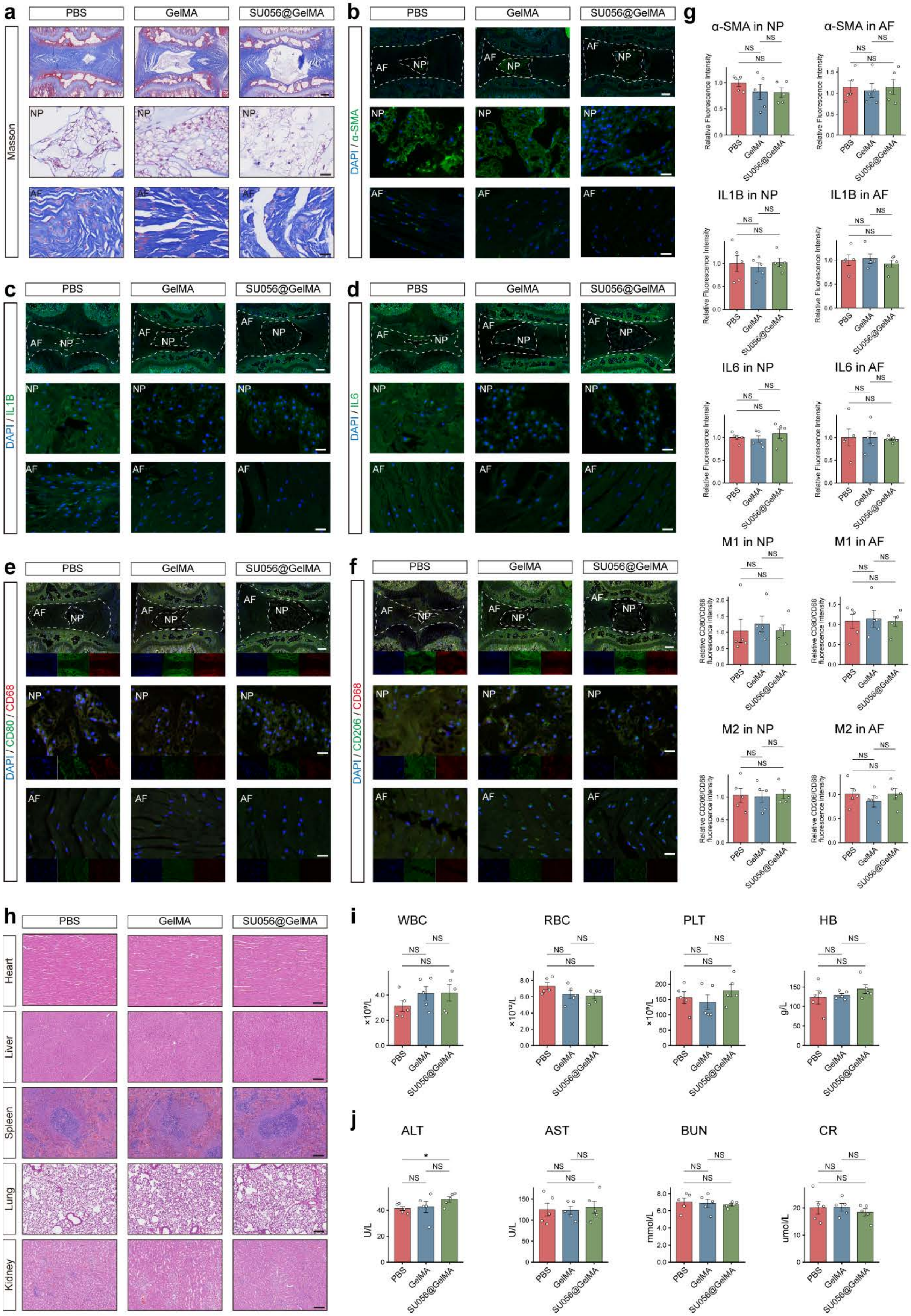
a
PBS
GelMA
SU056@GelMA
Masson
NP
AF
b
DAPI / α-SMA
c
DAPI / IL1B
d
DAPI / IL6
e
DAPI / CD80 / CD68
f
DAPI / CD206 / CD68
g
α-SMA in NP
α-SMA in AF
IL1B in NP
IL1B in AF
IL6 in NP
IL6 in AF
M1 in NP
M1 in AF
M2 in NP
M2 in AF
Relative Fluorescence Intensity
Relative CD80/CD68 fluorescence intensity
Relative CD206/CD68 fluorescence intensity
NS
h
Heart
Liver
Spleen
Lung
Kidney
i
WBC
RBC
PLT
HB
×10⁹/L
×10¹²/L
g/L
j
ALT
AST
BUN
CR
U/L
mmol/L
umol/L
*

Supplementary Fig. 8. Biosafety evaluation of SU056@GelMA. (a) Representative Masson's trichrome staining images of rat caudal intervertebral disc injection sites after intradiscal injection of PBS, GelMA, or SU056@GelMA. Magnified regions show the nucleus pulposus and annulus fibrosus. (b-d) Representative IF images of alpha-smooth muscle actin (alpha-SMA), interleukin-1beta (IL-1beta), and interleukin-6 (IL-6) at rat caudal disc injection sites after different treatments (n = 5 per group). Magnified regions show the nucleus pulposus and annulus fibrosus. (e, f) Representative IF images of CD68/CD80 and CD68/CD206 at rat caudal disc injection sites after different treatments (n = 5 per group). Magnified regions show the nucleus pulposus and annulus fibrosus. (g) Quantification of the IF images shown in b-f. (h) Representative H&E staining images of heart, liver, spleen, lung, and kidney after different treatments. (i) Quantification of complete blood count parameters, including white blood cells (WBC), red blood cells (RBC), platelets (PLT), and hemoglobin (HB), after different treatments (n = 5 per group). (j) Quantification of serum biochemical parameters, including alanine aminotransferase (ALT), aspartate aminotransferase (AST), blood urea nitrogen (BUN), and creatinine (Cr), after different treatments (n = 5 per group). Images within each panel were acquired using the same scale. Scale bars: 300 um for low-magnification images and 50 um for high-magnification images in Masson and IF images, and 200 um for H&E images.

Data are presented as mean ± SEM. Statistical significance was determined using two-tailed unpaired Welch's t-test for the indicated pairwise comparisons; NS, not significant; *P < 0.05, **P < 0.01, ***P < 0.001 (panels g, i and j).

Supplementary Table 1. Patients information

Patient	Gender	Age	Piffrmann Grade	Segment	Mean Age
Patient1	Female	54	II	L4/L5	
Patient2	Male	33	II	L4/L5-L5/S1	
Patient3	Male	61	II	L3/L4-L5/S1	
Patient4	Female	59	II	L3/L4-L5/S1	51
Patient5	Male	58	II	L3/4-L5/S1	
Patient6	Male	22	II	L4/L5	
Patient7	Female	71	II	L4/L5-L5/S1	
Patient8	Female	51	III	L1/2-L4/L5	
Patient9	Female	50	III	L3/L4-L5/S1	
Patient10	Female	47	III	L4/L5-L5/S1	
Patient11	Male	50	III	L4/L5-L5/S1	
Patient12	Male	23	III	L4/L5	49
Patient13	Male	38	III	L4/L5-L5/S1	
Patient14	Male	20	III	L4/L5-L5/S1	
Patient15	Male	69	III	L4/L5-L5/S1	
Patient16	Female	70	III	L1/2	
Patient17	Male	72	III	L2/3-L4/5	
Patient18	Male	83	IV	L2/3、L3/4、L4/5、L5/S1	
Patient19	Male	68	IV	L1/2-L4/L5	
Patient20	Female	41	IV	L4/5-L5/S1	
Patient21	Male	72	IV	L2/L3-L5/S1	
Patient22	Male	28	IV	L5/S1	
Patient23	Male	58	IV	L3/L4-L5/S1	58
Patient24	Male	59	IV	L3/L4-L5/S1	
Patient25	Female	46	IV	L4/L5-L5/S1	
Patient26	Male	59	IV	L4/L5-L5/S1	
Patient27	Male	58	IV	L4/L5-L5/S1	
Patient28	Male	80	IV	L1/2-L4/5	
Patient29	Male	44	IV	L4/L5-L5/S1	
Patient30	Male	60	IV	L2/3-L5/S1	
Patient31	Male	62	V	L5/S1	
Patient32	Female	60	V	L4/L5-L5/S1	
Patient33	Male	45	V	L5/S1	57
Patient34	Female	39	V	L5/S1	
Patient35	Female	78	V	L1/2-L5/S1	

Supplementary Table 2. Primers for RT-qPCR

ID	Name	sequence
1	H-ACTIN-F	AGAGCTACGAGCTGCCTGAC
2	H-ACTIN-R	AGCACTGTGTTGGCGTACAG
3	H-ADAMTS5-F	GGGCACTGGCTACTATGTGG
4	H-ADAMTS5-R	CGTCACAGCCAGTTCTCACA
5	H-COL2-F	CCAGATGACCTTCCTACGCC
6	H-COL2-R	TTCAGGGCAGTGTACGTGAAC
7	H-HDAC1-F	GGACGGACCGACTGACGGTA
8	H-HDAC1-R	GGTTCAAAGTTAAGAACGGGAAGAATG
9	H-HDAC2-F	TCCGCATGACCCATAACTTGC
10	H-HDAC2-R	CCGCCAGTTGAGAGCTGAC
11	H-HDAC3-F	GCAAGGCTTCACCAAGAGTCT
12	H-HDAC3-R	AGATGCGCCTGTGTAACGC
13	H-HDAC4-F	CCTGGGAATGTACGACGCC
14	H-HDAC4-R	CCCGTCTTTCCTGCGTAAC
15	H-HDAC5-F	GGTGTGGTCTACGACACGTTC
16	H-HDAC5-R	GATCCGCTCGCACTTGCTAA
17	H-HDAC6-F	AAGAAGACCTAATCGTGGGACT
18	H-HDAC6-R	GCTGTGAACCAACATCAGCTC
19	H-HDAC7-F	TGCCCAGTCCTTAATGACCAC
20	H-HDAC7-R	CACCTGGACGTGAGTTTTGAG
21	H-HDAC8-F	TCGCTGGTCCCGGTTTATATC
22	H-HDAC8-R	TACTGGCCCGTTTGGGGAT
23	H-HDAC9-F	ATGGTTTCACAGCAACGCATT
24	H-HDAC9-R	ACCTTGCCTAAGCGTCTGC
25	H-HDAC10-F	CAGTTCGACGCCATCTACTTC
26	H-HDAC10-R	CAAGCCCATTTTGCACAGCTC
27	H-HDAC11-F	CACGCTCGCCATCAAGTTTC
28	H-HDAC11-R	GAAGTCTCGCTCATGCCCATT
29	H-MMP3-F	CTGGACTCCGACACTCTGGA
30	H-MMP3-R	CAGGAAAGGTTCTGAAGTGACC
31	H-SOX9-F	GCTCTGGAGACTTCTGAACGA
32	H-SOX9-R	CCGTTCTTCACCGACTTCCT
33	H-TP53-F	CAGCACATGACGGAGGTTGT
34	H-TP53-R	TCATCCAAATACTCCACACGC
35	H-YB1-F	CCCCAGGAAGTACCTTCGC
36	H-YB1-R	AGCGTCTATAATGGTTACGGTCT
